# Supplementary material for: Evaluating patient factors, operative management and postoperative outcomes in trauma laparotomy patients worldwide: a protocol for a global observational multicentre trauma study
Source: BMJ Open. 2024 Apr 5;14(4):e083135. doi: 10.1136/bmjopen-2023-083135 (PMC11002395; doi:10.1136/bmjopen-2023-083135)
Supplement: Supplementary data [file bmjopen-2023-083135supp001.pdf]

Supplementary Material

Authorship Model

Fully named authors on the by-line of any publications resulting from this study will be those that satisfy the International Committee of Medical Journal Editors (www.icmje.org) guidelines for authorship, namely those in the Steering Committee and Advisory Committee.

All collaborators (Local Leads, Local Collaborators, and Data Validators) will then be listed individually as PubMed-citable collaborator status authors under the collaborator authorship name, as outlined in the Guidelines for Standardising Reporting of Authorship in Collaborative Research (23). The roles are defined below:

- Steering Committee - A core group who have the responsibility for protocol design, project co-ordination, and data handling
- Advisory Committee – An external group of doctors with expertise within trauma and pre-hospital care to provide advice and guidance to the development and completion of the study
- Local Lead – A doctor who ensures the successful delivery of the study at their respective centre; their responsibilities include:
    - Obtaining appropriate governance registration at their centre
    - Identifying, managing, and co-ordinating their centre's collaborators and data validator throughout the study
    - Providing point of contact for patient enquiries if required locally
    - Ensuring all data is uploaded to the secure web-based data capture system by the relevant deadline
- Local Collaborators – A group of up to 3 doctors, healthcare professionals, or medical students responsible for the data collection for each data collection period at their respective centre
- Data Validator – A doctor, healthcare professional, or medical student, independent to the data collection team, at select centres used to check case ascertainment and data accuracy in a select study period

Tables

Supplementary Table 1 – Data Collection Points for the GOAL-Trauma Study

**Admission**	
Age	
Sex	Male/Female/Unknown
Mechanism of Injury	Blunt/Penetrating
Date/Time of Injury	HH:MM DD/MM/YYYY
Date/Time of Presentation to Hospital	HH:MM DD/MM/YYYY
Date/Time of Decision to Operate	HH:MM DD/MM/YYYY
Was the patient directly transferred from the scene of injury to the hospital where surgery is taking place?	Yes / No
Method of transport to the hospital where surgery is taking place? (optional)	Land Ambulance / Bystander Support / Private Vehicle / Police / By Foot / Air Ambulance / Other (please specify)
Physiology on Arrival	
- Oxygen Saturation (sO2)	%
- Inspired Oxygen	%
- Respiratory rate	Breaths per minute
- Heart rate	Beats per minute
- Blood pressure (systolic / diastolic)	mmHg
- Glasgow Coma Score	/15
Admission blood tests (optional)	
Base excess	mmol/L
Lactate	mmol/L
Haemoglobin	g/L

Creatinine<br>INR<br><br>Blood products given pre-operatively<br><br>Total pre-operative resuscitation (optional)<br>- Crystalloids<br>- Colloids<br>- Packed red blood cells<br>- Fresh frozen plasma<br>- Platelets<br>- Cryoprecipitate<br>- Whole blood<br>- Tranexamic acid<br><br>Pre-operative antibiotics<br><br><br>Pre-operative investigations	mmol/L<br><br><br>Yes / No<br><br><br>mL<br>mL<br>units<br>units<br>pools<br>units<br>units<br>g<br><br>Yes / No<br>If Yes, before arrival to presenting hospital / before arrival to theatre / in theatre<br><br>Blood tests<br>Ultrasound scan (including FAST scan)<br>Plain Film Radiographs (X-ray)<br>Computerized Tomography (CT) scan<br>Magnetic resonance imaging (MRI) scan<br>Diagnostic Peritoneal Lavage (DPL) or Aspiration (DPA)
**Operative**	
Indication for surgery<br><br><br><br><br>ASA score	Control bleeding<br>Control contamination<br>Exploration<br>Other<br><br>I II III IV V

Date/Time Operation Started	HH:MM DD/MM/YYYY
Physiology at Induction<br>- Oxygen Saturation (sO2)<br>- Inspired Oxygen<br>- Respiratory rate<br>- Heart rate<br>- Blood pressure (systolic / diastolic)<br>- Glasgow Coma Score	 %<br>%<br>Breaths per minute<br>Beats per minute<br>mmHg<br>/15
Intubated	Yes / No<br>If Yes, before arrival to presenting hospital / before arrival to theatre / in theatre
Regional Anaesthesia	Yes / No<br>If yes, spinal anaesthesia / epidural anaesthesia / transversus abdominis plane (TAP) block
Was the patient requiring circulatory support at time of induction?	Yes / No
Abdominal Injuries Sustained	As per AIS dictionary
Non-abdominal Injuries Sustained	As per AIS dictionary
Procedures Performed during Laparotomy (tick all that apply)	Upper gastrointestinal procedure: Resection (stapled or tied off stomach or bowel) / Resection (with anastomosis) / Bypass procedure (no resection) / Primary repair<br><br>Small bowel procedure: Resection (stapled or tied off bowel) / Resection (with anastomosis) / Primary repair / Stoma formation<br><br>Lower gastrointestinal procedure: Resection (stapled or tied off bowel) / Resection (with anastomosis) / Primary repair / Stoma formation<br><br>Vascular procedure: Ligation / Bypass / Primary Repair / Shunt / Graft / Other (specify)

	Other procedure: Nephrectomy / Splenectomy / Pancreatectomy (partial or complete) / Cholecystectomy / Liver packing / Pelvic Packing / Bladder Repair / Other (specify)<br><br>Negative laparotomy / Nontherapeutic laparotomy
Estimated blood loss	ml
Method of abdominal closure	Primary Closure / Left Open (for planned relook)
Concurrent operations (region involved)	Head or Neck / Chest / Upper Limb / Lower Limb / Other (please specify)
Intra-operative death	Yes / No
Senior surgeon grade	Consultant or Attending General Surgeon / Registrar or Resident General Surgeon (in training) / Other Surgeon Speciality (in training) / Medically Qualified but not in Surgical Training / Not Medically Qualified Surgical Provider
Senior anaesthesia provider grade	Consultant or Attending Anaesthetist / Registrar or Resident Anaesthetist (in training) / Not Medically Qualified Anaesthesia Provider / Anaesthetic Administered by the Surgeon
**Outcome**	
Date of discharge (or death if died on index admission)	DD/MM/YYYY
Post-operative organ support required:<br>- Circulatory support<br>- Respiratory support<br>- Renal support	 Vasopressor or Inotropic Agents / Mechanical / None<br>Intubation & Ventilation / Positive Pressure Support / None<br>Renal replacement therapy / None
In-hospital mortality<br>If Yes, was this attributable to a reversible cause? (optional)	Yes / No<br>Yes / No

Modified Clavin-Dindo Classification Morbidity<br><br>Return to theatre for abdominal surgery during the same admission?<br>If Yes, on what post-operative day<br><br><br>Discharge destination	 <br>Yes/No<br>If yes, planned or unplanned<br><br><br><br>Transfer to Another Hospital / Transfer to Rehabilitation Unit / Usual Place of Residence / Remains in Hospital at Day 30 / Absconded / Died / Other (state)<br>If discharged to Usual Place of Residence, are there now increased care requirements than before admission?<br>If increased care requirements, has this been met by formal or informal arrangements?
Any comments on this patient journey	
