# Supplementary material for: Evaluating patient factors, operative management and postoperative outcomes in trauma laparotomy patients worldwide: a protocol for a global observational multicentre trauma study
Source: BMJ Open. 2024 Apr 5;14(4):e083135. doi: 10.1136/bmjopen-2023-083135 (PMC11002395; doi:10.1136/bmjopen-2023-083135)
Supplement: Author's manuscript [file bmjopen-2023-083135.draft_revisions.pdf]

BMJ Open is committed to open peer review. As part of this commitment we make the peer review history of every article we publish publicly available.

When an article is published we post the peer reviewers' comments and the authors' responses online. We also post the versions of the paper that were used during peer review. These are the versions that the peer review comments apply to.

The versions of the paper that follow are the versions that were submitted during the peer review process. They are not the versions of record or the final published versions. They should not be cited or distributed as the published version of this manuscript.

BMJ Open is an open access journal and the full, final, typeset and author-corrected version of record of the manuscript is available on our site with no access controls, subscription charges or pay-per-view fees (http://bmjopen.bmj.com).

If you have any questions on BMJ Open's open peer review process please email info.bmjopen@bmj.com

BMJ Open

Protocol for The GOAL-Trauma Study: Global Outcomes After Laparotomy for Trauma

Journal:	*BMJ Open*
Manuscript ID	bmjopen-2023-083135
Article Type:	Protocol
Date Submitted by the Author:	12-Dec-2023
Complete List of Authors:	Bath, Michael; University of Cambridge, International Health Systems Group, Department of Engineering<br>Kohler, Katharina; University of Cambridge, International Health Systems Group, Department of Engineering; Cambridge University Hospitals NHS Foundation Trust, Department of Anaesthesia<br>Hobbs, Laura; University of Cambridge, International Health Systems Group, Department of Engineering; East and North Hertfordshire NHS Trust, Department of Anaesthesia<br>Smith, Brandon; University of Cambridge, International Health Systems Group, Department of Engineering<br>Clarke, David; University of Cambridge, Division of Neurosurgery, Department of Clinical Neurosciences<br>Kwizera, Arthur; Makerere University, Department of Anesthesia<br>Perkins, Zane; Queen Mary University of London, Centre for Trauma Sciences, Blizard Institute; Barts Health NHS Trust, Major Trauma Service, Royal London Hospital<br>Marsden, Max; Queen Mary University of London, Centre for Trauma Sciences, Blizard Institute; Defence Medical Services, Academic Department of Military Surgery and Trauma, Research and Clinical Innovation<br>Davenport, Ross; Queen Mary University of London, Centre for Trauma Sciences, Blizard Institute; Barts Health NHS Trust, Major Trauma Service, Royal London Hospital<br>Davies, Justin; Cambridge University Hospitals NHS Foundation Trust, Cambridge Colorectal Unit, Addenbrooke's Hospital; University of Cambridge, Department of Surgery<br>Amoako, Joachim; Korle Bu Teaching Hospital, Department of Surgery; University of Ghana Medical School<br>Moonesinghe, S. Ramani; NHS England, National Clinical Director for Critical and Perioperative Care<br>Weiser, Thomas; Stanford University,<br>Leather, Andy; King's College London, School of Life Course and Population Sciences<br>Hardcastle, Timothy ; University of KwaZulu-Natal, Department of Surgical Sciences, Mandela School of Medicine (NRMSM); KwaZulu-Natal Department of Health, Trauma and Burns Unit, Inkosi Albert Luthuli Central Hospital<br>Naidoo, Ravi; Ngwelezana Hospital, Department of Surgery<br>Nördin, Yannick; Emergency Medical Care System (SAMU)<br>Conway Morris, Andrew; University of Cambridge, Division of Anaesthesia, Department of Medicine

Hutchinson, Peter; University of Cambridge, Division of Neurosurgery, Department of Clinical Neurosciences; NIHR Global Health Research Group on Acquired Brain and Spine Injury
Bashford, Tom; University of Cambridge, International Health Systems Group, Department of Engineering; University of Cambridge, Division of Anaesthesia

Keywords: SURGERY, Observational Study, Health Services

SCHOLARONE™ Manuscripts

Protocol for The GOAL-Trauma Study: Global Outcomes After Laparotomy for Trauma

MF Bath[1], K Kohler[1,2], L Hobbs[1,3], B Smith[1], D Clarke[4], A Kwizera[5], Z Perkins[6,7], M Marsden[6,8], R Davenport[6,7], J Davies[9,10], J Amoako[11,12], R Moonesinghe[13], TG Weiser[14], A Leather[15], T Hardcastle[16,17], R Naidoo[18], Y Nordín Servín[19], A Conway-Morris[20], P Hutchinson[4,21], T Bashford[1,2]

[1] International Health Systems group, Department of Engineering, University of Cambridge, UK
[2] Department of Anaesthesia, Cambridge University Hospitals NHS Foundation Trust, UK
[3] Department of Anaesthesia, East & North Hertfordshire NHS Trust, UK
[4] Division of Neurosurgery, Department of Clinical Neurosciences, University of Cambridge, UK
[5] Department of Anaesthesia, Makerere University, Kampala, Uganda
[6] Centre for Trauma Sciences, Blizard Institute, Queen Mary University of London, UK
[7] Major Trauma Service, Royal London Hospital, Bart's Health NHS Trust, London UK
[8] Academic Department of Military Surgery and Trauma, RCI, Birmingham, UK
[9] Cambridge Colorectal Unit, Addenbrooke's Hospital, Cambridge University Hospitals NHS Foundation Trust, UK
[10] Department of Surgery, University of Cambridge, Cambridge, UK
[11] Department of Surgery, Korle Bu Teaching Hospital, Accra, Ghana
[12] University of Ghana Medical School, Accra, Ghana
[13] National Clinical Director for Critical and Perioperative Care, NHS England, UK
[14] Department of Surgery, Stanford University, Palo Alto, California, United States
[15] School of Life Course and Population Sciences, King's College London, London, UK
[16] Trauma and Burns Unit, Inkosi Albert Luthuli Central Hospital, KwaZulu-Natal Department of Health, Durban, South Africa
[17] Department of Surgical Sciences, Mandela School of Medicine (NRMSM), University of KwaZulu-Natal, Durban, South Africa
[18] Department of Surgery, Ngwelezana Hospital, Empangeni, South Africa
[19] Emergency Medical Care System (SAMU), Jalisco State, Mexico
[20] Division of Anaesthesia, Department of Medicine, University of Cambridge, UK
[21] NIHR Global Health Research Group on Acquired Brain and Spine Injury, Cambridge, UK

Corresponding Author:
Mr Michael F. Bath MBChB(Hons) BSc(Hons) MRes MRCS; mb2583@cam.ac.uk

Keywords: Trauma Systems, Laparotomy, Emergency Surgery, Trauma Surgery, Global Health

Declarations:
Ethics approval – Cambridge Psychology Research Ethics Committee
Conflicts of Interest – nil

Funding: Engineering and Physical Sciences Research Council (EPSRC) & University of Cambridge

For peer review only

Study Sponsor: University of Cambridge

For peer review only

Abstract

Introduction

Trauma contributes the greatest loss of disability-adjusted life-years for adolescents and young adults worldwide. In the context of global abdominal trauma, the trauma laparotomy is the most commonly performed operation. However, variation likely exists in how these patients are managed and their subsequent outcomes, yet very little global data on the topic currently exists. The objective of the GOAL-Trauma study is to evaluate both patient and injury factors for those undergoing trauma laparotomy, their clinical management, and post-operative outcomes.

Methods

We describe a planned prospective multi-centre observational cohort study of patients undergoing trauma laparotomy. We will include patients of all ages who present to hospital with a blunt or penetrating injury and undergo a trauma laparotomy within 5 days of presentation to the treating centre. The study will collect system, patient, process, and outcome data, following patients up until 30 days post-operatively (or until discharge or death, whichever is first). Our sample size calculation suggests we will need to recruit 552 patients from approximately 150 recruiting centres.

Discussion

The GOAL-Trauma study will provide a global snapshot of the current management and outcomes for patients undergoing a trauma laparotomy. It will also provide insight into the variation seen in the time delays in receiving care, the disease and patient factors present, and patient outcomes. For current standards of trauma care to be improved worldwide, a greater understanding of the current state of trauma laparotomy care is paramount if appropriate interventions and targets are to be identified and implemented.

For peer review only

Strengths and Limitations of this Study

- The GOAL-Trauma Study global snapshot of current standards in care for patients undergoing trauma laparotomy, from time of injury to time of discharge
- The data set includes aspects of patient and injury factors that will provide data to understand barriers and key target areas to improve overall care and outcomes in trauma care
- A global panel of trauma experts has developed with protocol, with significant involvement from Patient and Public Involvement (PPI) and multiple pilot studies planned
- This study is purely observational, therefore no new treatment or management options are being analysed

For peer review only

Introduction

Trauma accounts for approximately 9% of all global deaths (1) and contributes the greatest loss of disability-adjusted life-years for adolescents and young adults worldwide (2). In the context of global abdominal trauma, both blunt and penetrating, the trauma laparotomy is the most commonly performed operation; it can be used both as a means to access identified injuries to organs within the abdomen and pelvis, such as life-threatening bleeding from vessels or solid organs, or contamination from hollow viscus injuries, or for exploratory diagnostic reasons, where cross-sectional imaging may not be easily feasible or available. While mortality rates following a trauma laparotomy are substantial, ranging between 8-40% (3–8), in recent years the role of damage control resuscitation has transformed trauma care, with significant reduction in mortality and morbidity to trauma patients being observed (9–11). However, the standard of care and variation in practice occurring globally for patients undergoing a trauma laparotomy remain relatively unknown.

Comprehensive trauma care is a complex system that includes multiple components, from the first response and prehospital care elements, to definitive facility-based care, and then final rehabilitation and recovery, all whilst interacting with multiple healthcare services and regional infrastructures (12). As such, ensuring optimal trauma care provision can prove difficult, especially where health systems are fragile or under-resourced. A trauma laparotomy is a key procedure within emergency surgical care (13), yet if global trauma care is to be improved and optimised, high quality and nuanced data of patients accessing the trauma system are needed, from across the whole trauma care pathway.

Variation likely exists worldwide in the management and outcomes of patients following trauma laparotomy (14), however at present limited data exist. While similar post-operative outcomes have been observed in major trauma centres in a high income setting (3), this likely is not representation on the global scale, alongside varying injury patterns (15). Indeed, variations in the epidemiology and management of traumatic brain injury have been noted on the international level (16). Ensuring adequate trauma laparotomy care globally is essential if trauma outcomes are to be improved, however a greater understanding of the current state of trauma laparotomy care is paramount if appropriate interventions and targets are to be identified.

The objective of this study is to evaluate both patient and injury factors for those undergoing trauma laparotomy, their clinical management, and post-operative outcomes.

For peer review only

Methods

Aims

The primary aim of this study is to describe global 30-day post-operative mortality rates for patients undergoing a trauma laparotomy.

The secondary aims of the study are:

(1) To describe the epidemiological characteristics (demographics, injury characteristics, baseline clinical characteristics, and surgical case mix) for patients undergoing a trauma laparotomy

(2) To describe the pre-operative, peri-operative, and post-operative processes of care for patients undergoing a trauma laparotomy

Study Design

We will conduct a prospective multi-centre observational cohort study of patients undergoing a trauma laparotomy. We will recruit centres through pre-existing research networks, utilising a snowballing technique to expand registration of centres. We will enrol eligible patients undergoing emergency trauma laparotomy over a consecutive 30 day period at individual centres during the study dates, with patients followed until discharge, death, or 30 days post-operatively, whichever comes first. All data will be submitted to a central study team by 30 days after the end of their respective data study period.

Any hospital worldwide that performs emergency trauma surgery will be eligible to participate, including both trauma centres and trauma units; a minimum of 1 case must be submitted by the centre during the 30 day study period to be eligible. Each centre's team will be comprised of a local study lead, with maximum 3 members of the local study team for each data collection period. Independent data validators will be also required for select centres.

The study will be overseen and managed by the study's Steering Committee, with external advice provided by the study's Advisory Committee (see Appendix). All authors have been involved in the study design and protocol write-up.

For peer review only

Patient Criteria

We will include patients of all ages who present to hospital with a blunt or penetrating injury and undergo a trauma laparotomy within 5 days of presentation to the treating centre (day 0 being time of presentation). A patient will be included for final analysis if >70% of the data points required have been recorded.

Exclusion criteria are:

- Patients undergoing a laparotomy ≥ 5 days (i.e. 120 hours) since presentation to the treating centre
- Any relook laparotomy, including transfers from another centre for further and / or definitive surgery
- Any laparoscopic (including laparoscopic converted to open), robotic, or image-guided procedures
- Patients who have been recently discharged from any hospital (including for non-trauma related admissions) and have represented within 30 days of discharge

Data Collection

We aim to collect system, patient, process, and outcome data. A data set will be collected on all patients undergoing a trauma laparotomy within the inclusion period (Table 1). The included data fields were based on work by similar studies and refined through iterative consultation with a global interdisciplinary consortium of clinicians involved in trauma care.

Data will be collected through access to patient records only at each centre, performed by members of the local study team. The patient will not be contacted directly in any capacity during their inpatient stay or after the study and no direct involvement in patient care will occur. Data will be collected directly onto a well-established secure web-based system, REDCap cloud (or recorded temporarily onto a hard copy Data Collection Form and uploaded to REDCap at a later date)

A local data validator (independent of the local study team) will be appointed at select participating centres to assess the data accuracy and ascertainment. They will independently assess case ascertainment and collect data on 2 variables, namely the date and nature of the operation. A pilot study will be run prior to the main data collection period at multiple centres to ensure feasibility in the methodology in multiple settings.

The data collected will include a pragmatic set of variables that allow the proposed research to be conducted, but minimise both risk of identification and exclude extraneous possibly sensitive medical information. Local requisite ethics and approvals will be applied for and be in place before any data

For peer review only

collection occurs. Access to the main data for analysis will be limited to named researchers from the Steering Committee, however collaborators at each centre will be able to access their own institutions data during the study period.

Statistical Analysis

A formal Statistical Analysis Plan will be produced. However, in brief, participating centres will be stratified based on their country into groups based on their Human Development Index (HDI) rank, with 30-day mortality then reported for each HDI group. Further analyses will include stratification against other economic and health indices, and multi-variable logistic modelling will be used to investigate primary and secondary study aims.

As there are a lack of data on trauma laparotomy globally, performing an accurate sample size calculation is not feasible. Data suggest that 30-day mortality rates following trauma laparotomy in high-income countries (HICs) are around 9% (3) and in LMICs are around 17% (17–19); using an alpha value = 0.05 and power = 80%, to show a difference between HIC and LMIC settings, a total of 552 patients are required to be recruited to the study. Based on data from other observational studies (3,7,8), we would expect over a 30-day observation period each centre to commit around 3-5 patients to the study. Therefore, with around 150 recruiting centres from our pre-existing research network, we would expect to meet the sample size required.

Missing values are to be expected in a study of this kind and will potentially affect certain centres more than others. To allow for potential imputation without introducing significant bias, we will include patients with ≥70% completeness. Multiple imputation methods will be used for the remaining missing data.

Ethical Approval

The study has received ethics approval from the Cambridge Psychology Research Ethics Committee. We have involved Patient and Public Involvement (PPI) groups in the development of both the study protocol and participant literature. As an observational study, no future amendments to the protocol are planned. The protocol has been written following SPIRIT guidelines (20).

According to the United Kingdom National Health Service (NHS) Health Research Authority tool (https://www.hra-decisiontools.org.uk/research/), the study can be considered locally as a clinical evaluation study in the United Kingdom (UK). Outside of the UK, each centre's local team will be responsible for obtaining the necessary local approvals in line with their hospital and / or national

For peer review only

regulations. Collaborators will need to confirm local approval has been granted at the time of uploading patient data.

The study will be registered to ClinicalTrials.gov and will be reported following the Strengthening the Reporting of Observational Studies in Epidemiology statement guidelines and checklists (21). The sponsor of the study will be the University of Cambridge, with support provided from a number of collaborators. Once complete, data and conclusions from the study will be disseminated through the research network to all collaborators, with all work from the study planned to be published in open-access journals.

For peer review only

Discussion

We present the protocol for a global observational multi-centre study aiming to evaluate the patient and injury factors in those undergoing a trauma laparotomy and to assess any variation observed in outcomes occurring globally. This study will provide a global snapshot of the current 30-day mortality rates following trauma laparotomy, as well as insight into the variation seen in the time delays in receiving care, the disease and patient factors present, and patient outcomes.

There remains a relative paucity of evidence regarding global outcomes following trauma laparotomy. Much of the current data on trauma laparotomy derives from the military setting, and the remaining civilian level data currently published predominantly comes from high-income settings. Without high quality data available, ensuring a greater awareness and development towards global trauma strategies is challenging. Trauma laparotomy is a vital procedure in trauma care, therefore this study will provide key insights into trauma management.

Trauma care is complex, with heterogenous patient groups and disease presentations, set within varying macro-healthcare and micro-clinical requirements (22). This study will collect data across the patient journey, allowing key limitations within this complex journey of trauma care to be identified. Data collection methods can often be challenging in LMIC settings, as robust numerical data may not be readily available, limiting conventional analysis techniques. We will utilise the Abbreviated Injury Scale (AIS) to categorise and quantify the wide spectrum and severity of traumatic injuries sustained (23). However, as with any trauma study, there remains a large selection bias, as there will be many unaccounted mortalities from trauma that did not make it to hospital, did not make it into theatre, or deemed not appropriate for surgery, therefore not included in the study. We also suspect there may be a relatively high amount of missing data, however we will attempt to adjust for this with multiple imputation methods.

This study will provide high quality observational multi-centre data on an important topic in trauma care globally. Identifying the key aspects of patient and injury factors in this patient cohort, both pre-hospital and in-hospital, will ensure the primary data required to understand barriers and key target areas to improve overall care and outcomes.

For peer review only

References

1. Murray CJL, Vos T, Lozano R, Naghavi M, Flaxman AD, Michaud C, et al. Disability-adjusted life years (DALYs) for 291 diseases and injuries in 21 regions, 1990–2010: a systematic analysis for the Global Burden of Disease Study 2010. The Lancet. 2012 Dec;380(9859):2197–223.

2. Vos T, Lim SS, Abbafati C, Abbas KM, Abbasi M, Abbasifard M, et al. Global burden of 369 diseases and injuries in 204 countries and territories, 1990–2019: a systematic analysis for the Global Burden of Disease Study 2019. The Lancet. 2020 Oct;396(10258):1204–22.

3. Marsden MER, Vulliamy PED, Carden R, Naumann DN, Davenport RA, National Trauma Research and Innovation Collaborative (NaTRIC). Trauma Laparotomy in the UK: A Prospective National Service Evaluation. J Am Coll Surg. 2021 Sep;233(3):383-394.e1.

4. Clarke JR, Trooskin SZ, Doshi PJ, Greenwald L, Mode CJ. Time to laparotomy for intra-abdominal bleeding from trauma does affect survival for delays up to 90 minutes. J Trauma. 2002 Mar;52(3):420–5.

5. Marsden M, Carden R, Navaratne L, Smith IM, Penn-Barwell JG, Kraven LM, et al. Outcomes following trauma laparotomy for hypotensive trauma patients: A UK military and civilian perspective. J Trauma Acute Care Surg. 2018 Sep;85(3):620–5.

6. Harvin JA, Maxim T, Inaba K, Martinez-Aguilar MA, King DR, Choudhry AJ, et al. Mortality after emergent trauma laparotomy. Journal of Trauma and Acute Care Surgery. 2017 Sep;83(3):464–8.

7. Bentin JM, Possfelt-Møller E, Svenningsen P, Rudolph SS, Sillesen M. A characterization of trauma laparotomies in a scandinavian setting: an observational study. Scand J Trauma Resusc Emerg Med. 2022 Dec 8;30(1):43.

8. Lee H, Kong V, Cheung C, Thirayan V, Rajaretnam N, Elsabagh A, et al. Trends in the Management of Abdominal Gunshot Wounds Over the Last Decade: A South African Experience. World J Surg. 2022 May 11;46(5):998–1005.

9. Cotton BA, Reddy N, Hatch QM, LeFebvre E, Wade CE, Kozar RA, et al. Damage Control Resuscitation Is Associated With a Reduction in Resuscitation Volumes and Improvement in Survival in 390 Damage Control Laparotomy Patients. Ann Surg. 2011 Oct;254(4):598–605.

10. Cole E, Weaver A, Gall L, West A, Nevin D, Tallach R, et al. A Decade of Damage Control Resuscitation. Ann Surg. 2021 Jun;273(6):1215–20.

For peer review only

11. Joseph B, Azim A, Zangbar B, Bauman Z, O'Keeffe T, Ibraheem K, et al. Improving mortality in trauma laparotomy through the evolution of damage control resuscitation. Journal of Trauma and Acute Care Surgery. 2017 Feb;82(2):328–33.

12. Bashford T, Clarkson PJ, Menon DK, Hutchinson PJA. Unpicking the Gordian knot: a systems approach to traumatic brain injury care in low-income and middle-income countries. BMJ Glob Health. 2018;3(2):e000768.

13. O'Neill KM, Greenberg SLM, Cherian M, Gillies RD, Daniels KM, Roy N, et al. Bellwether Procedures for Monitoring and Planning Essential Surgical Care in Low- and Middle-Income Countries: Caesarean Delivery, Laparotomy, and Treatment of Open Fractures. World J Surg. 2016 Nov 28;40(11):2611–9.

14. Roberts DJ, Faris PD, Ball CG, Kirkpatrick AW, Moore EE, Feliciano D V., et al. Variation in use of damage control laparotomy for trauma by trauma centers in the United States, Canada, and Australasia. World Journal of Emergency Surgery. 2021 Dec 14;16(1):53.

15. Traynor MD, Hernandez MC, Aho JM, Wise K, Kong V, Clarke D, et al. Damage Control Laparotomy: High-Volume Centers Display Similar Mortality Rates Despite Differences in Country Income Level. World J Surg. 2020 Dec 31;44(12):3993–8.

16. Clark D, Joannides A, Adeleye AO, Bajamal AH, Bashford T, Biluts H, et al. Casemix, management, and mortality of patients receiving emergency neurosurgery for traumatic brain injury in the Global Neurotrauma Outcomes Study: a prospective observational cohort study. Lancet Neurol. 2022 May;21(5):438–49.

17. Ayoade B, Salami B, Tade A, Musa A, Olawoye OA. Abdominal Injuries in Olabisi Onabanjo University Teaching Hospital Sagamu, Nigeria: Pattern and Outcome. Nigerian Journal of Orthopaedics And Trauma. 2006;5(2):45–9.

18. Naidoo R, Faurie M, Oosthuizen G, Hardcastle T. Comparative outcome analysis of trauma and non-trauma emergency laparotomy using a modified NELA tool format. South African Journal of Surgery. 2021;59(1).

19. Adenuga AT, Adeyeye A. Pattern of Presentation and Outcome of Adult Patients with Abdominal Trauma - A 7-Year Retrospective Study in a Nigerian Tertiary Hospital. J Emerg Trauma Shock. 2023;16(1):8–12.

For peer review only

20. Chan AW, Tetzlaff JM, Gøtzsche PC, Altman DG, Mann H, Berlin JA, et al. SPIRIT 2013 explanation and elaboration: guidance for protocols of clinical trials. BMJ. 2013 Jan 8;346:e7586.

21. von Elm E, Altman DG, Egger M, Pocock SJ, Gøtzsche PC, Vandenbroucke JP. The Strengthening the Reporting of Observational Studies in Epidemiology (STROBE) statement: guidelines for reporting observational studies. Lancet. 2007;

22. Odland ML, Abdul-Latif AM, Ignatowicz A, Alayande B, Appia Ofori B, Balanikas E, et al. Equitable access to quality trauma systems in low-income and middle-income countries: assessing gaps and developing priorities in Ghana, Rwanda and South Africa. BMJ Glob Health. 2022 Apr 11;7(4):e008256.

23. Association For The Advancement Of Automotive Medicine. Abbreviated Injury Scale [Internet]. [cited 2023 Jul 12]. Available from: https://www.aaam.org/abbreviated-injury-scale-ais/about-ais/

For peer review only

Tables

Table 1 – Data Collection Points for the GOAL-Trauma Study

Admission	
Age	
Sex	Male/Female/Unknown
Mechanism of Injury	Blunt/Penetrating
Date/Time of Injury	HH:MM DD/MM/YYYY
Date/Time of Presentation to Hospital	HH:MM DD/MM/YYYY
Date/Time of Decision to Operate	HH:MM DD/MM/YYYY
Was the patient directly transferred from the scene of injury to the hospital where surgery is taking place?	Yes / No
Method of transport to the hospital where surgery is taking place? (optional)	Land Ambulance / Air Ambulance / Private Vehicle / Police / By Foot / Other (please specify)
Physiology on Arrival	
- Oxygen Saturation (sO2)	%
- Inspired Oxygen	%
- Respiratory rate	Breaths per minute
- Heart rate	Beats per minute
- Blood pressure (systolic / diastolic)	mmHg
- Glasgow Coma Score	/15
Admission blood tests (optional)	
Base excess	mmol/L
Lactate	mmol/L

For peer review only

Haemoglobin<br>Creatinine<br>INR	g/L<br>mmol/L
Blood products given pre-operatively	Yes / No
Total pre-operative resuscitation (optional)<br>- Crystalloids<br>- Colloids<br>- Packed red blood cells<br>- Fresh frozen plasma<br>- Platelets<br>- Cryoprecipitate<br>- Whole blood<br>- Tranexamic acid	 mL<br>mL<br>units<br>units<br>pools<br>units<br>units<br>g
Pre-operative antibiotics	Yes / No<br>If Yes, before arrival to presenting hospital / before arrival to theatre / in theatre
Pre-operative investigations	Blood tests<br>Ultrasound scan (including FAST scan)<br>Plain Film Radiographs (X-ray)<br>Computerized Tomography (CT) scan<br>Magnetic resonance imaging (MRI) scan<br>Diagnostic Peritoneal Lavage (DPL) or Aspiration (DPA)
**Operative**	
Indication for surgery	Control bleeding<br>Control contamination<br>Exploration<br>Other
ASA score	I II III IV V

For peer review only

Date/Time Operation Started	HH:MM DD/MM/YYYY
Physiology at Induction<br>- Oxygen Saturation (sO2)<br>- Inspired Oxygen<br>- Respiratory rate<br>- Heart rate<br>- Blood pressure (systolic / diastolic)<br>- Glasgow Coma Score	 %<br>%<br>Breaths per minute<br>Beats per minute<br>mmHg<br>/15
Intubated	Yes / No<br>If Yes, before arrival to presenting hospital / before arrival to theatre / in theatre
Regional Anaesthesia	Yes / No<br>If yes, spinal anaesthesia / epidural anaesthesia / transversus abdominis plane (TAP) block
Was the patient requiring circulatory support at time of induction?	Yes / No
Abdominal Injuries Sustained	As per AIS dictionary
Non-abdominal Injuries Sustained	As per AIS dictionary
Procedures Performed during Laparotomy (tick all that apply)	Upper gastrointestinal procedure: Resection (stapled or tied off stomach or bowel) / Resection (with anastomosis) / Bypass procedure (no resection) / Primary repair<br><br>Small bowel procedure: Resection (stapled or tied off bowel) / Resection (with anastomosis) / Primary repair / Stoma formation<br><br>Lower gastrointestinal procedure: Resection (stapled or tied off bowel) / Resection (with anastomosis) / Primary repair / Stoma formation

For peer review only

	Vascular procedure: Ligation / Bypass / Primary Repair / Shunt / Graft / Other (specify)<br><br>Other procedure: Nephrectomy / Splenectomy / Pancreatectomy (partial or complete) / Cholecystectomy / Liver packing / Pelvic Packing / Bladder Repair / Other (specify)<br><br>Negative laparotomy / Nontherapeutic laparotomy
Estimated blood loss	ml
Method of abdominal closure	Primary Closure / Left Open (for planned relook)
Concurrent operations (region involved)	Head or Neck / Chest / Upper Limb / Lower Limb / Other (please specify)
Intra-operative death	Yes / No
Senior surgeon grade	Consultant or Attending General Surgeon / Registrar or Resident General Surgeon (in training) / Other Surgeon Speciality (in training) / Medically Qualified but not in Surgical Training / Not Medically Qualified Surgical Provider
Senior anaesthesia provider grade	Consultant or Attending Anaesthetist / Registrar or Resident Anaesthetist (in training) / Not Medically Qualified Anaesthesia Provider / Anaesthetic Administered by the Surgeon
**Outcome**	
Date of discharge (or death if died on index admission)	DD/MM/YYYY
Post-operative organ support required:<br>- Circulatory support<br>- Respiratory support<br>- Renal support	 Vasopressor or Inotropic Agents / Mechanical / None<br>Intubation & Ventilation / Positive Pressure Support / None<br>Renal replacement therapy / None
In-hospital mortality	Yes / No<br>Yes / No

For peer review only

If Yes, was this attributable to a reversible cause? (optional)<br><br>Modified Clavin-Dindo Classification Morbidity<br><br>Return to theatre for abdominal surgery during the same admission?<br>If Yes, on what post-operative day<br><br><br>Discharge destination	 <br><br><br>Yes/No<br>If yes, planned or unplanned<br>POD<br><br>Transfer to Another Hospital / Transfer to Rehabilitation Unit / Usual Place of Residence / Remains in Hospital at Day 30 / Absconded / Died / Other (state)
Any comments on this patient journey	

For peer review only

Supplementary Material

Authorship Model

Fully named authors on the by-line of any publications resulting from this study will be those that satisfy the International Committee of Medical Journal Editors (www.icmje.org) guidelines for authorship, namely those in the Steering Committee and Advisory Committee.

All collaborators (Local Leads, Local Collaborators, and Data Validators) will then be listed individually as PubMed-citable collaborator status authors under the collaborator authorship name, as outlined in the Guidelines for Standardising Reporting of Authorship in Collaborative Research (23). The roles are defined below:

- Steering Committee - A core group who have the responsibility for protocol design, project co-ordination, and data handling
- Advisory Committee – An external group of doctors with expertise within trauma and pre-hospital care to provide advice and guidance to the development and completion of the study
- Local Lead – A doctor who ensures the successful delivery of the study at their respective centre; their responsibilities include:
    - Obtaining appropriate governance registration at their centre
    - Identifying, managing, and co-ordinating their centre's collaborators and data validator throughout the study
    - Providing point of contact for patient enquiries if required locally
    - Ensuring all data is uploaded to the secure web-based data capture system by the relevant deadline
- Local Collaborators – A group of up to 3 doctors, healthcare professionals, or medical students responsible for the data collection for each data collection period at their respective centre
- Data Validator – A doctor, healthcare professional, or medical student, independent to the data collection team, at select centres used to check case ascertainment and data accuracy in a select study period

For peer review only

Reporting checklist for protocol of a clinical trial.

Based on the SPIRIT guidelines.

Instructions to authors

Complete this checklist by entering the page numbers from your manuscript where readers will find each of the items listed below.

Your article may not currently address all the items on the checklist. Please modify your text to include the missing information. If you are certain that an item does not apply, please write "n/a" and provide a short explanation.

Upload your completed checklist as an extra file when you submit to a journal.

In your methods section, say that you used the SPIRITreporting guidelines, and cite them as:

Chan A-W, Tetzlaff JM, Gøtzsche PC, Altman DG, Mann H, Berlin J, Dickersin K, Hróbjartsson A, Schulz KF, Parulekar WR, Krleža-Jerić K, Laupacis A, Moher D. SPIRIT 2013 Explanation and Elaboration: Guidance for protocols of clinical trials. BMJ. 2013;346:e7586

		Reporting Item	Page Number
**Administrative information**			
Title	#1	Descriptive title identifying the study design, population, interventions, and, if applicable, trial acronym	1
Trial registration	#2a	Trial identifier and registry name. If not yet registered, name of intended registry	6
Trial registration: data set	#2b	All items from the World Health Organization Trial Registration Data Set	n/a
Protocol version	#3	Date and version identifier	1
Funding	#4	Sources and types of financial, material, and other support	1
Roles and responsibilities: contributorship	#5a	Names, affiliations, and roles of protocol contributors	4

For peer review only

Roles and responsibilities: sponsor contact information	#5b	Name and contact information for the trial sponsor	1
Roles and responsibilities: sponsor and funder	#5c	Role of study sponsor and funders, if any, in study design; collection, management, analysis, and interpretation of data; writing of the report; and the decision to submit the report for publication, including whether they will have ultimate authority over any of these activities	1
Roles and responsibilities: committees	#5d	Composition, roles, and responsibilities of the coordinating centre, steering committee, endpoint adjudication committee, data management team, and other individuals or groups overseeing the trial, if applicable (see Item 21a for data monitoring committee)	4
**Introduction**			
Background and rationale	#6a	Description of research question and justification for undertaking the trial, including summary of relevant studies (published and unpublished) examining benefits and harms for each intervention	3
Background and rationale: choice of comparators	#6b	Explanation for choice of comparators	3
Objectives	#7	Specific objectives or hypotheses	3
Trial design	#8	Description of trial design including type of trial (eg, parallel group, crossover, factorial, single group), allocation ratio, and framework (eg, superiority, equivalence, non-inferiority, exploratory)	4
**Methods: Participants, interventions, and outcomes**			
Study setting	#9	Description of study settings (eg, community clinic, academic hospital) and list of countries where data will be collected. Reference to where list of study sites can be obtained	4

For peer review only

Eligibility criteria	#10	Inclusion and exclusion criteria for participants. If applicable, eligibility criteria for study centres and individuals who will perform the interventions (eg, surgeons, psychotherapists)	5
Interventions: description	#11a	Interventions for each group with sufficient detail to allow replication, including how and when they will be administered	4
Interventions: modifications	#11b	Criteria for discontinuing or modifying allocated interventions for a given trial participant (eg, drug dose change in response to harms, participant request, or improving / worsening disease)	n/a
Interventions: adherance	#11c	Strategies to improve adherence to intervention protocols, and any procedures for monitoring adherence (eg, drug tablet return; laboratory tests)	n/a
Interventions: concomitant care	#11d	Relevant concomitant care and interventions that are permitted or prohibited during the trial	n/a
Outcomes	#12	Primary, secondary, and other outcomes, including the specific measurement variable (eg, systolic blood pressure), analysis metric (eg, change from baseline, final value, time to event), method of aggregation (eg, median, proportion), and time point for each outcome. Explanation of the clinical relevance of chosen efficacy and harm outcomes is strongly recommended	12-16
Participant timeline	#13	Time schedule of enrolment, interventions (including any run-ins and washouts), assessments, and visits for participants. A schematic diagram is highly recommended (see Figure)	4
Sample size	#14	Estimated number of participants needed to achieve study objectives and how it was determined, including clinical and statistical assumptions supporting any sample size calculations	6
Recruitment	#15	Strategies for achieving adequate participant enrolment to reach target sample size	4
**Methods: Assignment of interventions (for controlled trials)**			
Allocation: sequence generation	#16a	Method of generating the allocation sequence (eg, computer-generated random numbers), and list of any factors for stratification. To reduce predictability of a random sequence, details of any planned restriction (eg, blocking) should be	n/a

For peer review only

		provided in a separate document that is unavailable to those who enrol participants or assign interventions	
Allocation concealment mechanism	#16b	Mechanism of implementing the allocation sequence (eg, central telephone; sequentially numbered, opaque, sealed envelopes), describing any steps to conceal the sequence until interventions are assigned	n/a
Allocation: implementation	#16c	Who will generate the allocation sequence, who will enrol participants, and who will assign participants to interventions	n/a
Blinding (masking)	#17a	Who will be blinded after assignment to interventions (eg, trial participants, care providers, outcome assessors, data analysts), and how	n/a
Blinding (masking): emergency unblinding	#17b	If blinded, circumstances under which unblinding is permissible, and procedure for revealing a participant's allocated intervention during the trial	n/a
**Methods: Data collection, management, and analysis**			
Data collection plan	#18a	Plans for assessment and collection of outcome, baseline, and other trial data, including any related processes to promote data quality (eg, duplicate measurements, training of assessors) and a description of study instruments (eg, questionnaires, laboratory tests) along with their reliability and validity, if known. Reference to where data collection forms can be found, if not in the protocol	5
Data collection plan: retention	#18b	Plans to promote participant retention and complete follow-up, including list of any outcome data to be collected for participants who discontinue or deviate from intervention protocols	n/a
Data management	#19	Plans for data entry, coding, security, and storage, including any related processes to promote data quality (eg, double data entry; range checks for data values). Reference to where details of data management procedures can be found, if not in the protocol	5
Statistics: outcomes	#20a	Statistical methods for analysing primary and secondary outcomes. Reference to where other details of the statistical analysis plan can be found, if not in the protocol	6

For peer review only

Statistics: additional analyses	#20b	Methods for any additional analyses (eg, subgroup and adjusted analyses)	6
Statistics: analysis population and missing data	#20c	Definition of analysis population relating to protocol non-adherence (eg, as randomised analysis), and any statistical methods to handle missing data (eg, multiple imputation)	6
**Methods: Monitoring**			
Data monitoring: formal committee	#21a	Composition of data monitoring committee (DMC); summary of its role and reporting structure; statement of whether it is independent from the sponsor and competing interests; and reference to where further details about its charter can be found, if not in the protocol. Alternatively, an explanation of why a DMC is not needed	n/a
Data monitoring: interim analysis	#21b	Description of any interim analyses and stopping guidelines, including who will have access to these interim results and make the final decision to terminate the trial	n/a
Harms	#22	Plans for collecting, assessing, reporting, and managing solicited and spontaneously reported adverse events and other unintended effects of trial interventions or trial conduct	n/a
Auditing	#23	Frequency and procedures for auditing trial conduct, if any, and whether the process will be independent from investigators and the sponsor	n/a
**Ethics and dissemination**			
Research ethics approval	#24	Plans for seeking research ethics committee / institutional review board (REC / IRB) approval	6
Protocol amendments	#25	Plans for communicating important protocol modifications (eg, changes to eligibility criteria, outcomes, analyses) to relevant parties (eg, investigators, REC / IRBs, trial participants, trial registries, journals, regulators)	6
Consent or assent	#26a	Who will obtain informed consent or assent from potential trial participants or authorised surrogates, and how (see Item 32)	n/a

For peer review only

Consent or assent: ancillary studies	#26b	Additional consent provisions for collection and use of participant data and biological specimens in ancillary studies, if applicable	n/a
Confidentiality	#27	How personal information about potential and enrolled participants will be collected, shared, and maintained in order to protect confidentiality before, during, and after the trial	5
Declaration of interests	#28	Financial and other competing interests for principal investigators for the overall trial and each study site	1
Data access	#29	Statement of who will have access to the final trial dataset, and disclosure of contractual agreements that limit such access for investigators	5
Ancillary and post trial care	#30	Provisions, if any, for ancillary and post-trial care, and for compensation to those who suffer harm from trial participation	n/a
Dissemination policy: trial results	#31a	Plans for investigators and sponsor to communicate trial results to participants, healthcare professionals, the public, and other relevant groups (eg, via publication, reporting in results databases, or other data sharing arrangements), including any publication restrictions	7
Dissemination policy: authorship	#31b	Authorship eligibility guidelines and any intended use of professional writers	4
Dissemination policy: reproducible research	#31c	Plans, if any, for granting public access to the full protocol, participant-level dataset, and statistical code	n/a
**Appendices**			
Informed consent materials	#32	Model consent form and other related documentation given to participants and authorised surrogates	n/a
Biological specimens	#33	Plans for collection, laboratory evaluation, and storage of biological specimens for genetic or molecular analysis in the current trial and for future use in ancillary studies, if applicable	n/a

The SPIRIT Explanation and Elaboration paper is distributed under the terms of the Creative Commons Attribution License CC-BY-NC. This checklist was completed on 12. December 2023 using https://www.goodreports.org/, a tool made by the EQUATOR Network in collaboration with Penelope.ai

For peer review only

BMJ Open

Evaluating Patient Factors, Operative Management, and Post-Operative Outcomes in Trauma Laparotomy Patients Worldwide - A Protocol for a Global Observational Multi-Centre Trauma Study

Journal:	*BMJ Open*
Manuscript ID	bmjopen-2023-083135.R1
Article Type:	Protocol
Date Submitted by the Author:	02-Mar-2024

Complete List of Authors:	Bath, Michael; University of Cambridge, International Health Systems Group, Department of Engineering<br>Kohler, Katharina; University of Cambridge, International Health Systems Group, Department of Engineering; Cambridge University Hospitals NHS Foundation Trust, Department of Anaesthesia<br>Hobbs, Laura; University of Cambridge, International Health Systems Group, Department of Engineering; East and North Hertfordshire NHS Trust, Department of Anaesthesia<br>Smith, Brandon; University of Cambridge, International Health Systems Group, Department of Engineering<br>Clark, David; University of Cambridge, Division of Neurosurgery, Department of Clinical Neurosciences<br>Kwizera, Arthur; Makerere University, Department of Anesthesia<br>Perkins, Zane; Queen Mary University of London, Centre for Trauma Sciences, Blizard Institute; Barts Health NHS Trust, Major Trauma Service, Royal London Hospital<br>Marsden, Max; Queen Mary University of London, Centre for Trauma Sciences, Blizard Institute; Defence Medical Services, Academic Department of Military Surgery and Trauma, Research and Clinical Innovation<br>Davenport, Ross; Queen Mary University of London, Centre for Trauma Sciences, Blizard Institute; Barts Health NHS Trust, Major Trauma Service, Royal London Hospital<br>Davies, Justin; Cambridge University Hospitals NHS Foundation Trust, Cambridge Colorectal Unit, Addenbrooke's Hospital; University of Cambridge, Department of Surgery<br>Amoako, Joachim; Korle Bu Teaching Hospital, Department of Surgery; University of Ghana Medical School<br>Moonesinghe, S. Ramani; NHS England, National Clinical Director for Critical and Perioperative Care<br>Weiser, Thomas; Stanford University,<br>Leather, Andy; King's College London, School of Life Course and Population Sciences<br>Hardcastle, Timothy ; University of KwaZulu-Natal, Department of Surgical Sciences, Mandela School of Medicine (NRMSM); KwaZulu-Natal Department of Health, Trauma and Burns Unit, Inkosi Albert Luthuli Central Hospital<br>Naidoo, Ravi; Ngwelezana Hospital, Department of Surgery

	Nördin, Yannick; Emergency Medical Care System (SAMU)<br>Conway Morris, Andrew; University of Cambridge, Division of Anaesthesia, Department of Medicine<br>Lakhoo, Kokila; University of Oxford, Department of Paediatric Surgery<br>Hutchinson, Peter; University of Cambridge, Division of Neurosurgery, Department of Clinical Neurosciences; NIHR Global Health Research Group on Acquired Brain and Spine Injury<br>Bashford, Tom; University of Cambridge, International Health Systems Group, Department of Engineering; University of Cambridge, Division of Anaesthesia

**Primary Subject Heading**:	Surgery
Secondary Subject Heading:	Global health, Public health
Keywords:	SURGERY, Observational Study, Health Services

SCHOLARONE™
Manuscripts

For peer review only - http://bmjopen.bmj.com/site/about/guidelines.xhtml

Evaluating Patient Factors, Operative Management, and Post-Operative Outcomes in Trauma Laparotomy Patients Worldwide - A Protocol for a Global Observational Multi-Centre Trauma Study

MF Bath[1], K Kohler[1,2], L Hobbs[1,3], B Smith[1], D Clark[4], A Kwizera[5], Z Perkins[6,7], M Marsden[6,8], R Davenport[6,7], J Davies[9,10], J Amoako[11,12], R Moonesinghe[13], TG Weiser[14], A Leather[15], T Hardcastle[16,17], R Naidoo[18], Y Nordín Servín[19], A Conway-Morris[20], K Lakhoo[21], P Hutchinson[4,22], T Bashford[1,2]

[1] International Health Systems group, Department of Engineering, University of Cambridge, UK
[2] Department of Anaesthesia, Cambridge University Hospitals NHS Foundation Trust, UK
[3] Department of Anaesthesia, East & North Hertfordshire NHS Trust, UK
[4] Division of Neurosurgery, Department of Clinical Neurosciences, University of Cambridge, UK
[5] Department of Anaesthesia, Makerere University, Kampala, Uganda
[6] Centre for Trauma Sciences, Blizard Institute, Queen Mary University of London, UK
[7] Major Trauma Service, Royal London Hospital, Barts Health NHS Trust, London UK
[8] Academic Department of Military Surgery and Trauma, RCI, Birmingham, UK
[9] Cambridge Colorectal Unit, Addenbrooke's Hospital, Cambridge University Hospitals NHS Foundation Trust, UK
[10] Department of Surgery, University of Cambridge, Cambridge, UK
[11] Department of Surgery, Korle Bu Teaching Hospital, Accra, Ghana
[12] University of Ghana Medical School, Accra, Ghana
[13] National Clinical Director for Critical and Perioperative Care, NHS England, UK
[14] Department of Surgery, Stanford University, Palo Alto, California, United States
[15] School of Life Course and Population Sciences, King's College London, London, UK
[16] Trauma and Burns Unit, Inkosi Albert Luthuli Central Hospital, KwaZulu-Natal Department of Health, Durban, South Africa
[17] Department of Surgical Sciences, Mandela School of Medicine (NRMSM), University of KwaZulu-Natal, Durban, South Africa
[18] Department of Surgery, Ngwelezana Hospital, Empangeni, South Africa
[19] Emergency Medical Care System (SAMU), Jalisco State, Mexico
[20] Division of Anaesthesia, Department of Medicine, University of Cambridge, UK
[21] Department of Paediatric Surgery, University of Oxford, Oxford, UK
[22] NIHR Global Health Research Group on Acquired Brain and Spine Injury, Cambridge, UK

Corresponding Author:
Mr Michael F. Bath MBChB(Hons) BSc(Hons) MRes MRCS; mb2583@cam.ac.uk

Keywords: Trauma Systems, Laparotomy, Emergency Surgery, Trauma Surgery, Global Health

For peer review only

Abstract

Introduction

Trauma contributes the greatest loss of disability-adjusted life-years for adolescents and young adults worldwide. In the context of global abdominal trauma, the trauma laparotomy is the most commonly performed operation. Variation likely exists in how these patients are managed and their subsequent outcomes, yet very little global data on the topic currently exists. The objective of the GOAL-Trauma study is to evaluate both patient and injury factors for those undergoing trauma laparotomy, their clinical management, and post-operative outcomes.

Methods

We describe a planned prospective multi-centre observational cohort study of patients undergoing trauma laparotomy. We will include patients of all ages who present to hospital with a blunt or penetrating injury and undergo a trauma laparotomy within 5 days of presentation to the treating centre. The study will collect system, patient, process, and outcome data, following patients up until 30 days post-operatively (or until discharge or death, whichever is first). Our sample size calculation suggests we will need to recruit 552 patients from approximately 150 recruiting centres.

Discussion

The GOAL-Trauma study will provide a global snapshot of the current management and outcomes for patients undergoing a trauma laparotomy. It will also provide insight into the variation seen in the time delays for receiving care, the disease and patient factors present, and patient outcomes. For current standards of trauma care to be improved worldwide, a greater understanding of the current state of trauma laparotomy care is paramount if appropriate interventions and targets are to be identified and implemented.

For peer review only

Strengths and Limitations of this Study

- The GOAL-Trauma Study will provide a global snapshot of current standards in care for patients undergoing trauma laparotomy, from time of injury to time of discharge
- The data set includes aspects of patient and injury factors that will provide improved understanding in the barriers and target areas to improve overall care and outcomes for trauma patients
- A global panel of trauma experts has developed with protocol, with significant involvement from Patient and Public Involvement (PPI) groups
- This study is purely observational, therefore no new treatment or management options are being analysed

For peer review only

Introduction

Trauma accounts for approximately 9% of all global deaths (1) and contributes the greatest loss of disability-adjusted life-years for adolescents and young adults worldwide (2). In the context of global abdominal trauma, both blunt and penetrating, the trauma laparotomy is the most commonly performed operation; it can be used both as a means to access identified injuries to organs within the abdomen and pelvis, such as life-threatening bleeding from vessels or solid organs, or contamination from hollow viscus injuries, or for exploratory diagnostic reasons, where cross-sectional imaging may not be easily feasible or available. While mortality rates following a trauma laparotomy are substantial, ranging between 8-40% (3–8), in recent years the role of damage control resuscitation has transformed trauma care, with significant reduction in mortality and morbidity to trauma patients being observed (9–11). However, the standard of care and variation in practice occurring globally for patients undergoing a trauma laparotomy remain relatively unknown.

Comprehensive trauma care is a complex system that includes multiple components, from the first response and prehospital care elements, to definitive facility-based care, and then final rehabilitation and recovery, all whilst interacting with multiple healthcare services and regional infrastructures (12). As such, ensuring optimal trauma care provision can prove difficult, especially where health systems are fragile or under-resourced. A trauma laparotomy is a key procedure within emergency surgical care (13), yet if global trauma care is to be improved and optimised, high quality and nuanced data of patients accessing the trauma system are needed, from across the whole trauma care pathway.

Variation likely exists worldwide in the management and outcomes of patients following trauma laparotomy (14), however at present limited data exist. While similar post-operative outcomes have been observed in major trauma centres in a high income setting (3), this likely is not representation on the global scale, alongside varying injury patterns (15). Indeed, variations in the epidemiology and management of traumatic brain injury have been noted on the international level (16). Ensuring adequate trauma laparotomy care globally is essential if trauma outcomes are to be improved, however a greater understanding of the current state of trauma laparotomy care is paramount if appropriate interventions and targets are to be identified.

The objective of this study is to evaluate both patient and injury factors for those undergoing trauma laparotomy, their clinical management, and post-operative outcomes.

For peer review only

Methods

Aims

The primary aim of this study is to describe global 30-day post-operative mortality rates for patients undergoing a trauma laparotomy.

The secondary aims of the study are:

(1) To describe the epidemiological characteristics (demographics, injury characteristics, baseline clinical characteristics, and surgical case mix) for patients undergoing a trauma laparotomy

(2) To describe the pre-operative, peri-operative, and post-operative processes of care for patients undergoing a trauma laparotomy

For peer review only

Study Design

We will conduct a prospective multi-centre observational cohort study of patients undergoing a trauma laparotomy. We will recruit centres through pre-existing research networks, utilising a snowballing technique to expand registration of centres. We will enrol eligible patients undergoing emergency trauma laparotomy over a consecutive 30 day period at individual centres during the study dates, with patients followed until discharge, death, or 30 days post-operatively, whichever comes first. All data will be submitted to a central study team by 30 days after the end of their respective data study period.

Any hospital worldwide that performs emergency trauma surgery will be eligible to participate, including both trauma centres and trauma units; a minimum of 1 case must be submitted by the centre during the 30 day study period to be eligible. Each centre's team will be comprised of a local study lead, with maximum 3 members of the local study team for each data collection period. Independent data validators will be also required for select centres. The data collection periods will run between 1st April 2024 and 31st December 2024, with final 30-day follow-up for the final patients expected to be completed on 30th January 2025.

The study will be overseen and managed by the study's Steering Committee, with external advice provided by the study's Advisory Committee (see Supplementary Material). All authors have been involved in the study design and protocol write-up.

Patient Criteria

We will include patients of all ages who present to hospital with a blunt or penetrating injury and undergo a trauma laparotomy within 5 days of presentation to the treating centre (day 0 being time of presentation). A patient will be included for final analysis if >70% of the data points required have been recorded.

Exclusion criteria are:

- Patients undergoing a laparotomy ≥ 5 days (i.e. 120 hours) since presentation to the treating centre
- Any relook laparotomy, including transfers from another centre for further and / or definitive surgery
- Any laparoscopic (including laparoscopic converted to open), robotic, or image-guided procedures
- Patients who have been recently discharged from any hospital (including for non-trauma related admissions) and have represented within 30 days of discharge

Data Collection

We aim to collect system, patient, process, and outcome data. A data set will be collected on all patients undergoing a trauma laparotomy within the inclusion period (see Supplementary Table 1). The included data fields were based on work by similar studies and refined through iterative consultation with a global interdisciplinary consortium of clinicians involved in trauma care.

Data will be collected through access to patient records only at each centre, performed by members of the local study team. The patient will not be contacted directly in any capacity during their inpatient stay or after the study and no direct involvement in patient care will occur. Data will be collected directly onto a well-established secure web-based system, REDCap cloud (or recorded temporarily onto a hard copy Data Collection Form and uploaded to REDCap at a later date).

A local data validator (independent of the local study team) will be appointed at select participating centres to assess the data accuracy and ascertainment. They will independently assess case ascertainment and collect data on 2 variables, namely the date and nature of the operation. Each centre's local lead will also be asked to complete a centre survey, to provide more background on their specific hospital settings, infrastructure, and capacity. A pilot study was run prior to the main data collection period at multiple centres globally to ensure feasibility in the methodology across multiple settings.

For peer review only

The data collected will include a pragmatic set of variables that allow the proposed research to be conducted, but minimise both risk of identification and exclude extraneous possibly sensitive medical information. Local requisite ethics and approvals will be applied for and be in place before any data collection occurs. The patient consent procedure will depend on the local and national guidance for the collaborating hospitals; specific guidance will be provided to collaborators on consenting if necessary and, where required, written consent forms will be obtained for patient inclusion in the study.

Access to the main data for analysis will be limited to named researchers from the Steering Committee, however collaborators at each centre will be able to access their own institutions data during the study period.

Statistical Analysis

A formal Statistical Analysis Plan will be produced. However, in brief, participating centres will be stratified based on their country into groups based on their Human Development Index (HDI) rank, with 30-day mortality then reported for each HDI group. Further analyses will include stratification against other economic and health indices, and multi-variable logistic modelling will be used to investigate primary and secondary study aims.

As there are a lack of data on trauma laparotomy globally, performing an accurate sample size calculation is not feasible. Data suggest that 30-day mortality rates following trauma laparotomy in high-income countries (HICs) are around 9% (3) and in LMICs are around 17% (17–19); using an alpha value = 0.05 and power = 80%, to show a difference between HIC and LMIC settings, a total of 552 patients are required to be recruited to the study. Based on data from other observational studies (3,7,8), we would expect over a 30-day observation period each centre to commit around 3-5 patients to the study. Therefore, with around 150 recruiting centres from our pre-existing research network, we would expect to meet the sample size required.

Missing values are to be expected in a study of this kind and will potentially affect certain centres more than others. To allow for potential imputation without introducing significant bias, we will include patients with ≥70% completeness. Multiple imputation methods will be used for the remaining missing data.

For peer review only

Ethical Approval

The study has received ethics approval from the Cambridge Psychology Research Ethics Committee (PRE.2023.119). According to the United Kingdom National Health Service (NHS) Health Research Authority tool (https://www.hra-decisiontools.org.uk/research/), the study can be considered locally as a clinical evaluation study in the United Kingdom (UK). Outside of the UK, each centre's local team will be responsible for obtaining the necessary local approvals in line with their hospital and / or national regulations. Collaborators will need to confirm local approval has been granted prior to of uploading patient data.

The study has been registered to ClinicalTrials.gov (NCT06180668) and will be reported following the Strengthening the Reporting of Observational Studies in Epidemiology statement guidelines and checklists (20). The sponsor of the study will be the University of Cambridge, with support provided from a number of collaborators. Once complete, data and conclusions from the study will be disseminated through the research network to all collaborators, with all work from the study planned to be published in open-access journals. As an observational study, no future amendments to the protocol are planned.

Patient and Public Involvement

We have involved Patient and Public Involvement (PPI) groups throughout the development of the study protocol and key participant literature.

For peer review only

Discussion

We present the protocol for a global observational multi-centre study aiming to evaluate the patient and injury factors in those undergoing a trauma laparotomy and to assess any variation observed in outcomes occurring globally. This study will provide a global snapshot of the current 30-day mortality rates following trauma laparotomy, as well as insight into the variation seen in the time delays in receiving trauma care, the disease and patient factors present, and patient outcomes.

There remains a relative paucity of evidence regarding global outcomes following trauma laparotomy. Much of the current data on trauma laparotomy derives from the military setting, and the remaining civilian level data currently published predominantly comes from high-income settings. Without high quality data available, ensuring a greater awareness and development towards global trauma strategies is challenging. Trauma laparotomy is a vital procedure in trauma care, therefore this study will provide key insights into trauma management.

Trauma care is complex, with heterogenous patient groups and disease presentations, set within varying macro-healthcare and micro-clinical requirements (21). This study will collect data across the patient journey, allowing key parts limitations within this complex journey of trauma care to be identified and unpicked. However, data collection methods can often be challenging in LMIC settings, as robust numerical data may not be readily available, limiting conventional analysis techniques. We will utilise the Abbreviated Injury Scale (AIS) to categorise and quantify the wide spectrum and severity of traumatic injuries sustained (22). However, as with any trauma study, there remains a large selection bias, as there will be many unaccounted mortalities from trauma that did not make it to hospital, did not make it into theatre, or deemed not appropriate for surgery, therefore not included in the study. We also suspect there may be a relatively high amount of missing data, however we will attempt to adjust for this with multiple imputation methods.

This study will provide high quality observational multi-centre data on an important topic in trauma care globally, using the trauma laparotomy as a surrogate marker. Identifying the key aspects of patient and injury factors in this patient cohort, both pre-hospital and in-hospital, will ensure the primary data required to understand barriers and key target areas to improve overall care and outcomes.

For peer review only

Declarations:
Ethics approval – Cambridge Psychology Research Ethics Committee
Conflicts of Interest – nil

Contributorship statement:
MFB and TB were involved in the conception of article
MFB, KK, LH, BS, DC, AK, ZP, MM, RD, JD, JA, RM, TGW, AL, TH, RN, YNS, ACM, KL, PH, and TB were all involved in the design and planning of the study, and in the drafting of the article
TB reviewed and finalised the article

Funding: Engineering and Physical Sciences Research Council (EPSRC) & University of Cambridge

Study Sponsor: University of Cambridge

For peer review only

References

1. Murray CJL, Vos T, Lozano R, Naghavi M, Flaxman AD, Michaud C, et al. Disability-adjusted life years (DALYs) for 291 diseases and injuries in 21 regions, 1990–2010: a systematic analysis for the Global Burden of Disease Study 2010. The Lancet. 2012 Dec;380(9859):2197–223.

2. Vos T, Lim SS, Abbafati C, Abbas KM, Abbasi M, Abbasifard M, et al. Global burden of 369 diseases and injuries in 204 countries and territories, 1990–2019: a systematic analysis for the Global Burden of Disease Study 2019. The Lancet. 2020 Oct;396(10258):1204–22.

3. Marsden MER, Vulliamy PED, Carden R, Naumann DN, Davenport RA, National Trauma Research and Innovation Collaborative (NaTRIC). Trauma Laparotomy in the UK: A Prospective National Service Evaluation. J Am Coll Surg. 2021 Sep;233(3):383-394.e1.

4. Clarke JR, Trooskin SZ, Doshi PJ, Greenwald L, Mode CJ. Time to laparotomy for intra-abdominal bleeding from trauma does affect survival for delays up to 90 minutes. J Trauma. 2002 Mar;52(3):420–5.

5. Marsden M, Carden R, Navaratne L, Smith IM, Penn-Barwell JG, Kraven LM, et al. Outcomes following trauma laparotomy for hypotensive trauma patients: A UK military and civilian perspective. J Trauma Acute Care Surg. 2018 Sep;85(3):620–5.

6. Harvin JA, Maxim T, Inaba K, Martinez-Aguilar MA, King DR, Choudhry AJ, et al. Mortality after emergent trauma laparotomy. Journal of Trauma and Acute Care Surgery. 2017 Sep;83(3):464–8.

7. Bentin JM, Possfelt-Møller E, Svenningsen P, Rudolph SS, Sillesen M. A characterization of trauma laparotomies in a scandinavian setting: an observational study. Scand J Trauma Resusc Emerg Med. 2022 Dec 8;30(1):43.

8. Lee H, Kong V, Cheung C, Thirayan V, Rajaretnam N, Elsabagh A, et al. Trends in the Management of Abdominal Gunshot Wounds Over the Last Decade: A South African Experience. World J Surg. 2022 May 11;46(5):998–1005.

9. Cotton BA, Reddy N, Hatch QM, LeFebvre E, Wade CE, Kozar RA, et al. Damage Control Resuscitation Is Associated With a Reduction in Resuscitation Volumes and Improvement in Survival in 390 Damage Control Laparotomy Patients. Ann Surg. 2011 Oct;254(4):598–605.

10. Cole E, Weaver A, Gall L, West A, Nevin D, Tallach R, et al. A Decade of Damage Control Resuscitation. Ann Surg. 2021 Jun;273(6):1215–20.

For peer review only

11. Joseph B, Azim A, Zangbar B, Bauman Z, O'Keeffe T, Ibraheem K, et al. Improving mortality in trauma laparotomy through the evolution of damage control resuscitation. Journal of Trauma and Acute Care Surgery. 2017 Feb;82(2):328–33.

12. Bashford T, Clarkson PJ, Menon DK, Hutchinson PJA. Unpicking the Gordian knot: a systems approach to traumatic brain injury care in low-income and middle-income countries. BMJ Glob Health. 2018;3(2):e000768.

13. O'Neill KM, Greenberg SLM, Cherian M, Gillies RD, Daniels KM, Roy N, et al. Bellwether Procedures for Monitoring and Planning Essential Surgical Care in Low- and Middle-Income Countries: Caesarean Delivery, Laparotomy, and Treatment of Open Fractures. World J Surg. 2016 Nov 28;40(11):2611–9.

14. Roberts DJ, Faris PD, Ball CG, Kirkpatrick AW, Moore EE, Feliciano D V., et al. Variation in use of damage control laparotomy for trauma by trauma centers in the United States, Canada, and Australasia. World Journal of Emergency Surgery. 2021 Dec 14;16(1):53.

15. Traynor MD, Hernandez MC, Aho JM, Wise K, Kong V, Clarke D, et al. Damage Control Laparotomy: High-Volume Centers Display Similar Mortality Rates Despite Differences in Country Income Level. World J Surg. 2020 Dec 31;44(12):3993–8.

16. Clark D, Joannides A, Adeleye AO, Bajamal AH, Bashford T, Biluts H, et al. Casemix, management, and mortality of patients receiving emergency neurosurgery for traumatic brain injury in the Global Neurotrauma Outcomes Study: a prospective observational cohort study. Lancet Neurol. 2022 May;21(5):438–49.

17. Ayoade B, Salami B, Tade A, Musa A, Olawoye OA. Abdominal Injuries in Olabisi Onabanjo University Teaching Hospital Sagamu, Nigeria: Pattern and Outcome. Nigerian Journal of Orthopaedics And Trauma. 2006;5(2):45–9.

18. Naidoo R, Faurie M, Oosthuizen G, Hardcastle T. Comparative outcome analysis of trauma and non-trauma emergency laparotomy using a modified NELA tool format. South African Journal of Surgery. 2021;59(1).

19. Adenuga AT, Adeyeye A. Pattern of Presentation and Outcome of Adult Patients with Abdominal Trauma - A 7-Year Retrospective Study in a Nigerian Tertiary Hospital. J Emerg Trauma Shock. 2023;16(1):8–12.

For peer review only

20. von Elm E, Altman DG, Egger M, Pocock SJ, Gøtzsche PC, Vandenbroucke JP. The Strengthening the Reporting of Observational Studies in Epidemiology (STROBE) statement: guidelines for reporting observational studies. Lancet. 2007;

21. Odland ML, Abdul-Latif AM, Ignatowicz A, Alayande B, Appia Ofori B, Balanikas E, et al. Equitable access to quality trauma systems in low-income and middle-income countries: assessing gaps and developing priorities in Ghana, Rwanda and South Africa. BMJ Glob Health. 2022 Apr 11;7(4):e008256.

22. Association For The Advancement Of Automotive Medicine. Abbreviated Injury Scale [Internet]. [cited 2023 Jul 12]. Available from: https://www.aaam.org/abbreviated-injury-scale-ais/about-ais/

For peer review only

Supplementary Material

Authorship Model

Fully named authors on the by-line of any publications resulting from this study will be those that satisfy the International Committee of Medical Journal Editors (www.icmje.org) guidelines for authorship, namely those in the Steering Committee and Advisory Committee.

All collaborators (Local Leads, Local Collaborators, and Data Validators) will then be listed individually as PubMed-citable collaborator status authors under the collaborator authorship name, as outlined in the Guidelines for Standardising Reporting of Authorship in Collaborative Research (23). The roles are defined below:

- Steering Committee - A core group who have the responsibility for protocol design, project co-ordination, and data handling
- Advisory Committee – An external group of doctors with expertise within trauma and pre-hospital care to provide advice and guidance to the development and completion of the study
- Local Lead – A doctor who ensures the successful delivery of the study at their respective centre; their responsibilities include:
  - Obtaining appropriate governance registration at their centre
  - Identifying, managing, and co-ordinating their centre's collaborators and data validator throughout the study
  - Providing point of contact for patient enquiries if required locally
  - Ensuring all data is uploaded to the secure web-based data capture system by the relevant deadline
- Local Collaborators – A group of up to 3 doctors, healthcare professionals, or medical students responsible for the data collection for each data collection period at their respective centre
- Data Validator – A doctor, healthcare professional, or medical student, independent to the data collection team, at select centres used to check case ascertainment and data accuracy in a select study period

For peer review only

Tables

Supplementary Table 1 – Data Collection Points for the GOAL-Trauma Study

**Admission**	
Age	
Sex	Male/Female/Unknown
Mechanism of Injury	Blunt/Penetrating
Date/Time of Injury	HH:MM DD/MM/YYYY
Date/Time of Presentation to Hospital	HH:MM DD/MM/YYYY
Date/Time of Decision to Operate	HH:MM DD/MM/YYYY
Was the patient directly transferred from the scene of injury to the hospital where surgery is taking place?	Yes / No
Method of transport to the hospital where surgery is taking place? (optional)	Land Ambulance / Bystander Support / Private Vehicle / Police / By Foot / Air Ambulance / Other (please specify)
Physiology on Arrival	
- Oxygen Saturation (sO2)	%
- Inspired Oxygen	%
- Respiratory rate	Breaths per minute
- Heart rate	Beats per minute
- Blood pressure (systolic / diastolic)	mmHg
- Glasgow Coma Score	/15
Admission blood tests (optional)	
Base excess	mmol/L
Lactate	mmol/L
Haemoglobin	g/L

For peer review only

Creatinine<br>INR	mmol/L
Blood products given pre-operatively	Yes / No
Total pre-operative resuscitation (optional)<br>- Crystalloids<br>- Colloids<br>- Packed red blood cells<br>- Fresh frozen plasma<br>- Platelets<br>- Cryoprecipitate<br>- Whole blood<br>- Tranexamic acid	 mL<br>mL<br>units<br>units<br>pools<br>units<br>units<br>g
Pre-operative antibiotics	Yes / No<br>If Yes, before arrival to presenting hospital / before arrival to theatre / in theatre
Pre-operative investigations	Blood tests<br>Ultrasound scan (including FAST scan)<br>Plain Film Radiographs (X-ray)<br>Computerized Tomography (CT) scan<br>Magnetic resonance imaging (MRI) scan<br>Diagnostic Peritoneal Lavage (DPL) or Aspiration (DPA)
**Operative**	
Indication for surgery	Control bleeding<br>Control contamination<br>Exploration<br>Other
ASA score	I II III IV V

For peer review only

Date/Time Operation Started	HH:MM DD/MM/YYYY
Physiology at Induction<br>- Oxygen Saturation (sO2)<br>- Inspired Oxygen<br>- Respiratory rate<br>- Heart rate<br>- Blood pressure (systolic / diastolic)<br>- Glasgow Coma Score	 %<br>%<br>Breaths per minute<br>Beats per minute<br>mmHg<br>/15
Intubated	Yes / No<br>If Yes, before arrival to presenting hospital / before arrival to theatre / in theatre
Regional Anaesthesia	Yes / No<br>If yes, spinal anaesthesia / epidural anaesthesia / transversus abdominis plane (TAP) block
Was the patient requiring circulatory support at time of induction?	Yes / No
Abdominal Injuries Sustained	As per AIS dictionary
Non-abdominal Injuries Sustained	As per AIS dictionary
Procedures Performed during Laparotomy (tick all that apply)	Upper gastrointestinal procedure: Resection (stapled or tied off stomach or bowel) / Resection (with anastomosis) / Bypass procedure (no resection) / Primary repair<br><br>Small bowel procedure: Resection (stapled or tied off bowel) / Resection (with anastomosis) / Primary repair / Stoma formation<br><br>Lower gastrointestinal procedure: Resection (stapled or tied off bowel) / Resection (with anastomosis) / Primary repair / Stoma formation<br><br>Vascular procedure: Ligation / Bypass / Primary Repair / Shunt / Graft / Other (specify)

For peer review only

	Other procedure: Nephrectomy / Splenectomy / Pancreatectomy (partial or complete) / Cholecystectomy / Liver packing / Pelvic Packing / Bladder Repair / Other (specify)<br><br>Negative laparotomy / Nontherapeutic laparotomy
Estimated blood loss	ml
Method of abdominal closure	Primary Closure / Left Open (for planned relook)
Concurrent operations (region involved)	Head or Neck / Chest / Upper Limb / Lower Limb / Other (please specify)
Intra-operative death	Yes / No
Senior surgeon grade	Consultant or Attending General Surgeon / Registrar or Resident General Surgeon (in training) / Other Surgeon Speciality (in training) / Medically Qualified but not in Surgical Training / Not Medically Qualified Surgical Provider
Senior anaesthesia provider grade	Consultant or Attending Anaesthetist / Registrar or Resident Anaesthetist (in training) / Not Medically Qualified Anaesthesia Provider / Anaesthetic Administered by the Surgeon
**Outcome**	
Date of discharge (or death if died on index admission)	DD/MM/YYYY
Post-operative organ support required:<br>- Circulatory support<br>- Respiratory support<br>- Renal support	 Vasopressor or Inotropic Agents / Mechanical / None<br>Intubation & Ventilation / Positive Pressure Support / None<br>Renal replacement therapy / None
In-hospital mortality<br>If Yes, was this attributable to a reversible cause? (optional)	Yes / No<br>Yes / No

For peer review only

Modified Clavin-Dindo Classification Morbidity<br><br>Return to theatre for abdominal surgery during the same admission?<br>If Yes, on what post-operative day<br><br><br>Discharge destination	 <br>Yes/No<br>If yes, planned or unplanned<br><br><br><br>Transfer to Another Hospital / Transfer to Rehabilitation Unit / Usual Place of Residence / Remains in Hospital at Day 30 / Absconded / Died / Other (state)<br>If discharged to Usual Place of Residence, are there now increased care requirements than before admission?<br>If increased care requirements, has this been met by formal or informal arrangements?
Any comments on this patient journey	

For peer review only

BMJ Open

Evaluating Patient Factors, Operative Management, and Post-Operative Outcomes in Trauma Laparotomy Patients Worldwide - A Protocol for a Global Observational Multi-Centre Trauma Study

Journal:	*BMJ Open*
Manuscript ID	bmjopen-2023-083135.R2
Article Type:	Protocol
Date Submitted by the Author:	04-Mar-2024
Complete List of Authors:	Bath, Michael; University of Cambridge, International Health Systems Group, Department of Engineering<br>Kohler, Katharina; University of Cambridge, International Health Systems Group, Department of Engineering; Cambridge University Hospitals NHS Foundation Trust, Department of Anaesthesia<br>Hobbs, Laura; University of Cambridge, International Health Systems Group, Department of Engineering; East and North Hertfordshire NHS Trust, Department of Anaesthesia<br>Smith, Brandon; University of Cambridge, International Health Systems Group, Department of Engineering<br>Clark, David; University of Cambridge, Division of Neurosurgery, Department of Clinical Neurosciences<br>Kwizera, Arthur; Makerere University, Department of Anesthesia<br>Perkins, Zane; Queen Mary University of London, Centre for Trauma Sciences, Blizard Institute; Barts Health NHS Trust, Major Trauma Service, Royal London Hospital<br>Marsden, Max; Queen Mary University of London, Centre for Trauma Sciences, Blizard Institute; Defence Medical Services, Academic Department of Military Surgery and Trauma, Research and Clinical Innovation<br>Davenport, Ross; Queen Mary University of London, Centre for Trauma Sciences, Blizard Institute; Barts Health NHS Trust, Major Trauma Service, Royal London Hospital<br>Davies, Justin; Cambridge University Hospitals NHS Foundation Trust, Cambridge Colorectal Unit, Addenbrooke's Hospital; University of Cambridge, Department of Surgery<br>Amoako, Joachim; Korle Bu Teaching Hospital, Department of Surgery; University of Ghana Medical School<br>Moonesinghe, S. Ramani; NHS England, National Clinical Director for Critical and Perioperative Care<br>Weiser, Thomas; Stanford University,<br>Leather, Andy; King's College London, School of Life Course and Population Sciences<br>Hardcastle, Timothy ; University of KwaZulu-Natal, Department of Surgical Sciences, Mandela School of Medicine (NRMSM); KwaZulu-Natal Department of Health, Trauma and Burns Unit, Inkosi Albert Luthuli Central Hospital<br>Naidoo, Ravi; Ngwelezana Hospital, Department of Surgery

	Nördin, Yannick; Emergency Medical Care System (SAMU)<br>Conway Morris, Andrew; University of Cambridge, Division of Anaesthesia, Department of Medicine<br>Lakhoo, Kokila; University of Oxford, Department of Paediatric Surgery<br>Hutchinson, Peter; University of Cambridge, Division of Neurosurgery, Department of Clinical Neurosciences; NIHR Global Health Research Group on Acquired Brain and Spine Injury<br>Bashford, Tom; University of Cambridge, International Health Systems Group, Department of Engineering; University of Cambridge, Division of Anaesthesia
**Primary Subject Heading**:	Surgery
Secondary Subject Heading:	Global health, Public health
Keywords:	SURGERY, Observational Study, Health Services

SCHOLARONE™
Manuscripts

Evaluating Patient Factors, Operative Management, and Post-Operative Outcomes in Trauma Laparotomy Patients Worldwide - A Protocol for a Global Observational Multi-Centre Trauma Study

MF Bath[1], K Kohler[1,2], L Hobbs[1,3], B Smith[1], D Clark[4], A Kwizera[5], Z Perkins[6,7], M Marsden[6,8], R Davenport[6,7], J Davies[9,10], J Amoako[11,12], R Moonesinghe[13], TG Weiser[14], A Leather[15], T Hardcastle[16,17], R Naidoo[18], Y Nordín Servín[19], A Conway-Morris[20], K Lakhoo[21], P Hutchinson[4,22], T Bashford[1,2]

[1] International Health Systems group, Department of Engineering, University of Cambridge, UK
[2] Department of Anaesthesia, Cambridge University Hospitals NHS Foundation Trust, UK
[3] Department of Anaesthesia, East & North Hertfordshire NHS Trust, UK
[4] Division of Neurosurgery, Department of Clinical Neurosciences, University of Cambridge, UK
[5] Department of Anaesthesia, Makerere University, Kampala, Uganda
[6] Centre for Trauma Sciences, Blizard Institute, Queen Mary University of London, UK
[7] Major Trauma Service, Royal London Hospital, Barts Health NHS Trust, London UK
[8] Academic Department of Military Surgery and Trauma, RCI, Birmingham, UK
[9] Cambridge Colorectal Unit, Addenbrooke's Hospital, Cambridge University Hospitals NHS Foundation Trust, UK
[10] Department of Surgery, University of Cambridge, Cambridge, UK
[11] Department of Surgery, Korle Bu Teaching Hospital, Accra, Ghana
[12] University of Ghana Medical School, Accra, Ghana
[13] National Clinical Director for Critical and Perioperative Care, NHS England, UK
[14] Department of Surgery, Stanford University, Palo Alto, California, United States
[15] School of Life Course and Population Sciences, King's College London, London, UK
[16] Trauma and Burns Unit, Inkosi Albert Luthuli Central Hospital, KwaZulu-Natal Department of Health, Durban, South Africa
[17] Department of Surgical Sciences, Mandela School of Medicine (NRMSM), University of KwaZulu-Natal, Durban, South Africa
[18] Department of Surgery, Ngwelezana Hospital, Empangeni, South Africa
[19] Emergency Medical Care System (SAMU), Jalisco State, Mexico
[20] Division of Anaesthesia, Department of Medicine, University of Cambridge, UK
[21] Department of Paediatric Surgery, University of Oxford, Oxford, UK
[22] NIHR Global Health Research Group on Acquired Brain and Spine Injury, Cambridge, UK

Corresponding Author:
Mr Michael F. Bath MBChB(Hons) BSc(Hons) MRes MRCS; mb2583@cam.ac.uk

Keywords: Trauma Systems, Laparotomy, Emergency Surgery, Trauma Surgery, Global Health

For peer review only

Abstract

Introduction

Trauma contributes the greatest loss of disability-adjusted life-years for adolescents and young adults worldwide. In the context of global abdominal trauma, the trauma laparotomy is the most commonly performed operation. Variation likely exists in how these patients are managed and their subsequent outcomes, yet very little global data on the topic currently exists. The objective of the GOAL-Trauma study is to evaluate both patient and injury factors for those undergoing trauma laparotomy, their clinical management, and post-operative outcomes.

Methods

We describe a planned prospective multi-centre observational cohort study of patients undergoing trauma laparotomy. We will include patients of all ages who present to hospital with a blunt or penetrating injury and undergo a trauma laparotomy within 5 days of presentation to the treating centre. The study will collect system, patient, process, and outcome data, following patients up until 30 days post-operatively (or until discharge or death, whichever is first). Our sample size calculation suggests we will need to recruit 552 patients from approximately 150 recruiting centres.

Discussion

The GOAL-Trauma study will provide a global snapshot of the current management and outcomes for patients undergoing a trauma laparotomy. It will also provide insight into the variation seen in the time delays for receiving care, the disease and patient factors present, and patient outcomes. For current standards of trauma care to be improved worldwide, a greater understanding of the current state of trauma laparotomy care is paramount if appropriate interventions and targets are to be identified and implemented.

For peer review only

Strengths and Limitations of this Study

- The GOAL-Trauma Study will provide a global snapshot of current standards in care for patients undergoing trauma laparotomy, from time of injury to time of discharge
- The data set includes aspects of patient and injury factors that will provide improved understanding in the barriers and target areas to improve overall care and outcomes for trauma patients
- A global panel of trauma experts has developed with protocol, with significant involvement from Patient and Public Involvement (PPI) groups
- This study is purely observational, therefore no new treatment or management options are being analysed

For peer review only

Introduction

Trauma accounts for approximately 9% of all global deaths (1) and contributes the greatest loss of disability-adjusted life-years for adolescents and young adults worldwide (2). In the context of global abdominal trauma, both blunt and penetrating, the trauma laparotomy is the most commonly performed operation; it can be used both as a means to access identified injuries to organs within the abdomen and pelvis, such as life-threatening bleeding from vessels or solid organs, or contamination from hollow viscus injuries, or for exploratory diagnostic reasons, where cross-sectional imaging may not be easily feasible or available. While mortality rates following a trauma laparotomy are substantial, ranging between 8-40% (3–8), in recent years the role of damage control resuscitation has transformed trauma care, with significant reduction in mortality and morbidity to trauma patients being observed (9–11). However, the standard of care and variation in practice occurring globally for patients undergoing a trauma laparotomy remain relatively unknown.

Comprehensive trauma care is a complex system that includes multiple components, from the first response and prehospital care elements, to definitive facility-based care, and then final rehabilitation and recovery, all whilst interacting with multiple healthcare services and regional infrastructures (12). As such, ensuring optimal trauma care provision can prove difficult, especially where health systems are fragile or under-resourced. A trauma laparotomy is a key procedure within emergency surgical care (13), yet if global trauma care is to be improved and optimised, high quality and nuanced data of patients accessing the trauma system are needed, from across the whole trauma care pathway.

Variation likely exists worldwide in the management and outcomes of patients following trauma laparotomy (14), however at present limited data exist. While similar post-operative outcomes have been observed in major trauma centres in a high income setting (3), this likely is not representation on the global scale, alongside varying injury patterns (15). Indeed, variations in the epidemiology and management of traumatic brain injury have been noted on the international level (16). Ensuring adequate trauma laparotomy care globally is essential if trauma outcomes are to be improved, however a greater understanding of the current state of trauma laparotomy care is paramount if appropriate interventions and targets are to be identified.

The objective of this study is to evaluate both patient and injury factors for those undergoing trauma laparotomy, their clinical management, and post-operative outcomes.

For peer review only

Methods

Aims

The primary aim of this study is to describe global 30-day post-operative mortality rates for patients undergoing a trauma laparotomy.

The secondary aims of the study are:

(1) To describe the epidemiological characteristics (demographics, injury characteristics, baseline clinical characteristics, and surgical case mix) for patients undergoing a trauma laparotomy

(2) To describe the pre-operative, peri-operative, and post-operative processes of care for patients undergoing a trauma laparotomy

Study Design

We will conduct a prospective multi-centre observational cohort study of patients undergoing a trauma laparotomy. We will recruit centres through pre-existing research networks, utilising a snowballing technique to expand registration of centres. We will enrol eligible patients undergoing emergency trauma laparotomy over a consecutive 30 day period at individual centres during the study dates, with patients followed until discharge, death, or 30 days post-operatively, whichever comes first. All data will be submitted to a central study team by 30 days after the end of their respective data study period.

Any hospital worldwide that performs emergency trauma surgery will be eligible to participate, including both trauma centres and trauma units; a minimum of 1 case must be submitted by the centre during the 30 day study period to be eligible. Each centre's team will be comprised of a local study lead, with maximum 3 members of the local study team for each data collection period. Independent data validators will be also required for select centres. The data collection periods will run between 1st April 2024 and 31st December 2024, with final 30-day follow-up for the final patients expected to be completed on 30th January 2025.

The study will be overseen and managed by the study's Steering Committee, with external advice provided by the study's Advisory Committee (see Supplementary Material). All authors have been involved in the study design and protocol write-up.

For peer review only

Patient Criteria

We will include patients of all ages who present to hospital with a blunt or penetrating injury and undergo a trauma laparotomy within 5 days of presentation to the treating centre (day 0 being time of presentation). A patient will be included for final analysis if >70% of the data points required have been recorded.

Exclusion criteria are:

- Patients undergoing a laparotomy ≥ 5 days (i.e. 120 hours) since presentation to the treating centre
- Any relook laparotomy, including transfers from another centre for further and / or definitive surgery
- Any laparoscopic (including laparoscopic converted to open), robotic, or image-guided procedures
- Patients who have been recently discharged from any hospital (including for non-trauma related admissions) and have represented within 30 days of discharge

Data Collection

We aim to collect system, patient, process, and outcome data. A data set will be collected on all patients undergoing a trauma laparotomy within the inclusion period (see Supplementary Table 1). The included data fields were based on work by similar studies and refined through iterative consultation with a global interdisciplinary consortium of clinicians involved in trauma care.

Data will be collected through access to patient records only at each centre, performed by members of the local study team. The patient will not be contacted directly in any capacity during their inpatient stay or after the study and no direct involvement in patient care will occur. Data will be collected directly onto a well-established secure web-based system, REDCap cloud (or recorded temporarily onto a hard copy Data Collection Form and uploaded to REDCap at a later date).

A local data validator (independent of the local study team) will be appointed at select participating centres to assess the data accuracy and ascertainment. They will independently assess case ascertainment and collect data on 2 variables, namely the date and nature of the operation. Each centre's local lead will also be asked to complete a centre survey, to provide more background on their specific hospital settings, infrastructure, and capacity. A pilot study was run prior to the main data collection period at multiple centres globally to ensure feasibility in the methodology across multiple settings.

For peer review only

The data collected will include a pragmatic set of variables that allow the proposed research to be conducted, but minimise both risk of identification and exclude extraneous possibly sensitive medical information. Local requisite ethics and approvals will be applied for and be in place before any data collection occurs. The patient consent procedure will depend on the local and national guidance for the collaborating hospitals; specific guidance will be provided to collaborators on consenting if necessary and, where required, written consent forms will be obtained for patient inclusion in the study.

Access to the main data for analysis will be limited to named researchers from the Steering Committee, however collaborators at each centre will be able to access their own institutions data during the study period.

Statistical Analysis

A formal Statistical Analysis Plan will be produced. However, in brief, participating centres will be stratified based on their country into groups based on their Human Development Index (HDI) rank, with 30-day mortality then reported for each HDI group. Further analyses will include stratification against other economic and health indices, and multi-variable logistic modelling will be used to investigate primary and secondary study aims.

As there are a lack of data on trauma laparotomy globally, performing an accurate sample size calculation is not feasible. Data suggest that 30-day mortality rates following trauma laparotomy in high-income countries (HICs) are around 9% (3) and in LMICs are around 17% (17–19); using an alpha value = 0.05 and power = 80%, to show a difference between HIC and LMIC settings, a total of 552 patients are required to be recruited to the study. Based on data from other observational studies (3,7,8), we would expect over a 30-day observation period each centre to commit around 3-5 patients to the study. Therefore, with around 150 recruiting centres from our pre-existing research network, we would expect to meet the sample size required.

Missing values are to be expected in a study of this kind and will potentially affect certain centres more than others. To allow for potential imputation without introducing significant bias, we will include patients with ≥70% completeness. Multiple imputation methods will be used for the remaining missing data.

For peer review only

Ethical Approval

The study has received ethics approval from the Cambridge Psychology Research Ethics Committee (PRE.2023.119). According to the United Kingdom National Health Service (NHS) Health Research Authority tool (https://www.hra-decisiontools.org.uk/research/), the study can be considered locally as a clinical evaluation study in the United Kingdom (UK). Outside of the UK, each centre's local team will be responsible for obtaining the necessary local approvals in line with their hospital and / or national regulations. Collaborators will need to confirm local approval has been granted prior to of uploading patient data.

The study has been registered to ClinicalTrials.gov (NCT06180668) and will be reported following the Strengthening the Reporting of Observational Studies in Epidemiology statement guidelines and checklists (20). The sponsor of the study will be the University of Cambridge, with support provided from a number of collaborators. Once complete, data and conclusions from the study will be disseminated through the research network to all collaborators, with all work from the study planned to be published in open-access journals. As an observational study, no future amendments to the protocol are planned.

Patient and Public Involvement

Patient and Public Involvement (PPI) groups were involved in the development of the study protocol and key participant literature. During the protocol development stage, a PPI discussion group was convened, involving with people who have specific lived experiences relevant to the project; feedback obtained from this session directly influenced aspects of methodology and proposed outcomes for the study. A separate PPI group further reviewed the proposed patient-focused literature associated with the study, providing comments and edits to this work, which were implemented in the eventual patient information sheets produced.

For peer review only

Discussion

We present the protocol for a global observational multi-centre study aiming to evaluate the patient and injury factors in those undergoing a trauma laparotomy and to assess any variation observed in outcomes occurring globally. This study will provide a global snapshot of the current 30-day mortality rates following trauma laparotomy, as well as insight into the variation seen in the time delays in receiving trauma care, the disease and patient factors present, and patient outcomes.

There remains a relative paucity of evidence regarding global outcomes following trauma laparotomy. Much of the current data on trauma laparotomy derives from the military setting, and the remaining civilian level data currently published predominantly comes from high-income settings. Without high quality data available, ensuring a greater awareness and development towards global trauma strategies is challenging. Trauma laparotomy is a vital procedure in trauma care, therefore this study will provide key insights into trauma management.

Trauma care is complex, with heterogenous patient groups and disease presentations, set within varying macro-healthcare and micro-clinical requirements (21). This study will collect data across the patient journey, allowing key parts limitations within this complex journey of trauma care to be identified and unpicked. However, data collection methods can often be challenging in LMIC settings, as robust numerical data may not be readily available, limiting conventional analysis techniques. We will utilise the Abbreviated Injury Scale (AIS) to categorise the wide spectrum and severity of traumatic injuries sustained (22), with the individual AIS scores further used to calculate an Injury Severity Score (ISS) for each patient, quantifying overall injury burden and will be adjusted for in the final data analysis.

As with any trauma study, there remains a large selection bias, as there will be many unaccounted mortalities from trauma that did not make it to hospital, did not make it into theatre, or deemed not appropriate for surgery, therefore not included in the study. We also suspect there may be a relatively high amount of missing data, however we will attempt to adjust for this with multiple imputation methods. Of further note, we are not including patients who have undergone any laparoscopic procedure (including laparoscopic converted to open), alongside any robotic procedures or interventional radiology procedures, to ensure the core data set provides the clearest picture on trauma provision and pathways globally.

The GOAL-Trauma study will provide high quality observational multi-centre data on an important topic in trauma care globally, using the trauma laparotomy as a surrogate marker. Identifying the key aspects of patient and injury factors in this patient cohort, both pre-hospital and in-hospital, will

For peer review only

ensure the primary data required to understand barriers and key target areas to improve overall care and outcomes.

For peer review only

Declarations:
Ethics approval – Cambridge Psychology Research Ethics Committee
Conflicts of Interest – nil

Contributorship statement:
MFB and TB were involved in the conception of article
MFB, KK, LH, BS, DC, AK, ZP, MM, RD, JD, JA, RM, TGW, AL, TH, RN, YNS, ACM, KL, PH, and TB were all involved in the design and planning of the study, and in the drafting of the article
TB reviewed and finalised the article

Funding: Engineering and Physical Sciences Research Council (EPSRC) & University of Cambridge

Study Sponsor: University of Cambridge

For peer review only

References

1. Murray CJL, Vos T, Lozano R, Naghavi M, Flaxman AD, Michaud C, et al. Disability-adjusted life years (DALYs) for 291 diseases and injuries in 21 regions, 1990–2010: a systematic analysis for the Global Burden of Disease Study 2010. The Lancet. 2012 Dec;380(9859):2197–223.

2. Vos T, Lim SS, Abbafati C, Abbas KM, Abbasi M, Abbasifard M, et al. Global burden of 369 diseases and injuries in 204 countries and territories, 1990–2019: a systematic analysis for the Global Burden of Disease Study 2019. The Lancet. 2020 Oct;396(10258):1204–22.

3. Marsden MER, Vulliamy PED, Carden R, Naumann DN, Davenport RA, National Trauma Research and Innovation Collaborative (NaTRIC). Trauma Laparotomy in the UK: A Prospective National Service Evaluation. J Am Coll Surg. 2021 Sep;233(3):383-394.e1.

4. Clarke JR, Trooskin SZ, Doshi PJ, Greenwald L, Mode CJ. Time to laparotomy for intra-abdominal bleeding from trauma does affect survival for delays up to 90 minutes. J Trauma. 2002 Mar;52(3):420–5.

5. Marsden M, Carden R, Navaratne L, Smith IM, Penn-Barwell JG, Kraven LM, et al. Outcomes following trauma laparotomy for hypotensive trauma patients: A UK military and civilian perspective. J Trauma Acute Care Surg. 2018 Sep;85(3):620–5.

6. Harvin JA, Maxim T, Inaba K, Martinez-Aguilar MA, King DR, Choudhry AJ, et al. Mortality after emergent trauma laparotomy. Journal of Trauma and Acute Care Surgery. 2017 Sep;83(3):464–8.

7. Bentin JM, Possfelt-Møller E, Svenningsen P, Rudolph SS, Sillesen M. A characterization of trauma laparotomies in a scandinavian setting: an observational study. Scand J Trauma Resusc Emerg Med. 2022 Dec 8;30(1):43.

8. Lee H, Kong V, Cheung C, Thirayan V, Rajaretnam N, Elsabagh A, et al. Trends in the Management of Abdominal Gunshot Wounds Over the Last Decade: A South African Experience. World J Surg. 2022 May 11;46(5):998–1005.

9. Cotton BA, Reddy N, Hatch QM, LeFebvre E, Wade CE, Kozar RA, et al. Damage Control Resuscitation Is Associated With a Reduction in Resuscitation Volumes and Improvement in Survival in 390 Damage Control Laparotomy Patients. Ann Surg. 2011 Oct;254(4):598–605.

10. Cole E, Weaver A, Gall L, West A, Nevin D, Tallach R, et al. A Decade of Damage Control Resuscitation. Ann Surg. 2021 Jun;273(6):1215–20.

For peer review only

11. Joseph B, Azim A, Zangbar B, Bauman Z, O'Keeffe T, Ibraheem K, et al. Improving mortality in trauma laparotomy through the evolution of damage control resuscitation. Journal of Trauma and Acute Care Surgery. 2017 Feb;82(2):328–33.

12. Bashford T, Clarkson PJ, Menon DK, Hutchinson PJA. Unpicking the Gordian knot: a systems approach to traumatic brain injury care in low-income and middle-income countries. BMJ Glob Health. 2018;3(2):e000768.

13. O'Neill KM, Greenberg SLM, Cherian M, Gillies RD, Daniels KM, Roy N, et al. Bellwether Procedures for Monitoring and Planning Essential Surgical Care in Low- and Middle-Income Countries: Caesarean Delivery, Laparotomy, and Treatment of Open Fractures. World J Surg. 2016 Nov 28;40(11):2611–9.

14. Roberts DJ, Faris PD, Ball CG, Kirkpatrick AW, Moore EE, Feliciano D V., et al. Variation in use of damage control laparotomy for trauma by trauma centers in the United States, Canada, and Australasia. World Journal of Emergency Surgery. 2021 Dec 14;16(1):53.

15. Traynor MD, Hernandez MC, Aho JM, Wise K, Kong V, Clarke D, et al. Damage Control Laparotomy: High-Volume Centers Display Similar Mortality Rates Despite Differences in Country Income Level. World J Surg. 2020 Dec 31;44(12):3993–8.

16. Clark D, Joannides A, Adeleye AO, Bajamal AH, Bashford T, Biluts H, et al. Casemix, management, and mortality of patients receiving emergency neurosurgery for traumatic brain injury in the Global Neurotrauma Outcomes Study: a prospective observational cohort study. Lancet Neurol. 2022 May;21(5):438–49.

17. Ayoade B, Salami B, Tade A, Musa A, Olawoye OA. Abdominal Injuries in Olabisi Onabanjo University Teaching Hospital Sagamu, Nigeria: Pattern and Outcome. Nigerian Journal of Orthopaedics And Trauma. 2006;5(2):45–9.

18. Naidoo R, Faurie M, Oosthuizen G, Hardcastle T. Comparative outcome analysis of trauma and non-trauma emergency laparotomy using a modified NELA tool format. South African Journal of Surgery. 2021;59(1).

19. Adenuga AT, Adeyeye A. Pattern of Presentation and Outcome of Adult Patients with Abdominal Trauma - A 7-Year Retrospective Study in a Nigerian Tertiary Hospital. J Emerg Trauma Shock. 2023;16(1):8–12.

For peer review only

20. von Elm E, Altman DG, Egger M, Pocock SJ, Gøtzsche PC, Vandenbroucke JP. The Strengthening the Reporting of Observational Studies in Epidemiology (STROBE) statement: guidelines for reporting observational studies. Lancet. 2007;

21. Odland ML, Abdul-Latif AM, Ignatowicz A, Alayande B, Appia Ofori B, Balanikas E, et al. Equitable access to quality trauma systems in low-income and middle-income countries: assessing gaps and developing priorities in Ghana, Rwanda and South Africa. BMJ Glob Health. 2022 Apr 11;7(4):e008256.

22. Association For The Advancement Of Automotive Medicine. Abbreviated Injury Scale [Internet]. [cited 2023 Jul 12]. Available from: https://www.aaam.org/abbreviated-injury-scale-ais/about-ais/

For peer review only

Supplementary Material

Authorship Model

Fully named authors on the by-line of any publications resulting from this study will be those that satisfy the International Committee of Medical Journal Editors (www.icmje.org) guidelines for authorship, namely those in the Steering Committee and Advisory Committee.

All collaborators (Local Leads, Local Collaborators, and Data Validators) will then be listed individually as PubMed-citable collaborator status authors under the collaborator authorship name, as outlined in the Guidelines for Standardising Reporting of Authorship in Collaborative Research (23). The roles are defined below:

- Steering Committee - A core group who have the responsibility for protocol design, project co-ordination, and data handling
- Advisory Committee – An external group of doctors with expertise within trauma and pre-hospital care to provide advice and guidance to the development and completion of the study
- Local Lead – A doctor who ensures the successful delivery of the study at their respective centre; their responsibilities include:
    - Obtaining appropriate governance registration at their centre
    - Identifying, managing, and co-ordinating their centre's collaborators and data validator throughout the study
    - Providing point of contact for patient enquiries if required locally
    - Ensuring all data is uploaded to the secure web-based data capture system by the relevant deadline
- Local Collaborators – A group of up to 3 doctors, healthcare professionals, or medical students responsible for the data collection for each data collection period at their respective centre
- Data Validator – A doctor, healthcare professional, or medical student, independent to the data collection team, at select centres used to check case ascertainment and data accuracy in a select study period

For peer review only

Tables

Supplementary Table 1 – Data Collection Points for the GOAL-Trauma Study

**Admission**	
Age	
Sex	Male/Female/Unknown
Mechanism of Injury	Blunt/Penetrating
Date/Time of Injury	HH:MM DD/MM/YYYY
Date/Time of Presentation to Hospital	HH:MM DD/MM/YYYY
Date/Time of Decision to Operate	HH:MM DD/MM/YYYY
Was the patient directly transferred from the scene of injury to the hospital where surgery is taking place?	Yes / No
Method of transport to the hospital where surgery is taking place? (optional)	Land Ambulance / Bystander Support / Private Vehicle / Police / By Foot / Air Ambulance / Other (please specify)
Physiology on Arrival	
- Oxygen Saturation (sO2)	%
- Inspired Oxygen	%
- Respiratory rate	Breaths per minute
- Heart rate	Beats per minute
- Blood pressure (systolic / diastolic)	mmHg
- Glasgow Coma Score	/15
Admission blood tests (optional)	
Base excess	mmol/L
Lactate	mmol/L
Haemoglobin	g/L

For peer review only

Creatinine<br>INR	mmol/L
Blood products given pre-operatively	Yes / No
Total pre-operative resuscitation (optional)<br>- Crystalloids<br>- Colloids<br>- Packed red blood cells<br>- Fresh frozen plasma<br>- Platelets<br>- Cryoprecipitate<br>- Whole blood<br>- Tranexamic acid	 mL<br>mL<br>units<br>units<br>pools<br>units<br>units<br>g
Pre-operative antibiotics	Yes / No<br>If Yes, before arrival to presenting hospital / before arrival to theatre / in theatre
Pre-operative investigations	Blood tests<br>Ultrasound scan (including FAST scan)<br>Plain Film Radiographs (X-ray)<br>Computerized Tomography (CT) scan<br>Magnetic resonance imaging (MRI) scan<br>Diagnostic Peritoneal Lavage (DPL) or Aspiration (DPA)
**Operative**	
Indication for surgery	Control bleeding<br>Control contamination<br>Exploration<br>Other
ASA score	I II III IV V

For peer review only

Date/Time Operation Started	HH:MM DD/MM/YYYY
Physiology at Induction<br>- Oxygen Saturation (sO2)<br>- Inspired Oxygen<br>- Respiratory rate<br>- Heart rate<br>- Blood pressure (systolic / diastolic)<br>- Glasgow Coma Score	 %<br>%<br>Breaths per minute<br>Beats per minute<br>mmHg<br>/15
Intubated	Yes / No<br>If Yes, before arrival to presenting hospital / before arrival to theatre / in theatre
Regional Anaesthesia	Yes / No<br>If yes, spinal anaesthesia / epidural anaesthesia / transversus abdominis plane (TAP) block
Was the patient requiring circulatory support at time of induction?	Yes / No
Abdominal Injuries Sustained	As per AIS dictionary
Non-abdominal Injuries Sustained	As per AIS dictionary
Procedures Performed during Laparotomy (tick all that apply)	Upper gastrointestinal procedure: Resection (stapled or tied off stomach or bowel) / Resection (with anastomosis) / Bypass procedure (no resection) / Primary repair<br><br>Small bowel procedure: Resection (stapled or tied off bowel) / Resection (with anastomosis) / Primary repair / Stoma formation<br><br>Lower gastrointestinal procedure: Resection (stapled or tied off bowel) / Resection (with anastomosis) / Primary repair / Stoma formation<br><br>Vascular procedure: Ligation / Bypass / Primary Repair / Shunt / Graft / Other (specify)

For peer review only

	Other procedure: Nephrectomy / Splenectomy / Pancreatectomy (partial or complete) / Cholecystectomy / Liver packing / Pelvic Packing / Bladder Repair / Other (specify)<br><br>Negative laparotomy / Nontherapeutic laparotomy
Estimated blood loss	ml
Method of abdominal closure	Primary Closure / Left Open (for planned relook)
Concurrent operations (region involved)	Head or Neck / Chest / Upper Limb / Lower Limb / Other (please specify)
Intra-operative death	Yes / No
Senior surgeon grade	Consultant or Attending General Surgeon / Registrar or Resident General Surgeon (in training) / Other Surgeon Speciality (in training) / Medically Qualified but not in Surgical Training / Not Medically Qualified Surgical Provider
Senior anaesthesia provider grade	Consultant or Attending Anaesthetist / Registrar or Resident Anaesthetist (in training) / Not Medically Qualified Anaesthesia Provider / Anaesthetic Administered by the Surgeon
**Outcome**	
Date of discharge (or death if died on index admission)	DD/MM/YYYY
Post-operative organ support required:<br>- Circulatory support<br>- Respiratory support<br>- Renal support	 Vasopressor or Inotropic Agents / Mechanical / None<br>Intubation & Ventilation / Positive Pressure Support / None<br>Renal replacement therapy / None
In-hospital mortality<br>If Yes, was this attributable to a reversible cause? (optional)	Yes / No<br>Yes / No

For peer review only

Modified Clavin-Dindo Classification Morbidity<br><br>Return to theatre for abdominal surgery during the same admission?<br>If Yes, on what post-operative day<br><br>Discharge destination	 <br>Yes/No<br>If yes, planned or unplanned<br><br>Transfer to Another Hospital / Transfer to Rehabilitation Unit / Usual Place of Residence / Remains in Hospital at Day 30 / Absconded / Died / Other (state)<br>If discharged to Usual Place of Residence, are there now increased care requirements than before admission?<br>If increased care requirements, has this been met by formal or informal arrangements?
Any comments on this patient journey	

For peer review only
